# Advances in Thermal Image Analysis for the Detection of Pregnancy in Horses Using Infrared Thermography

**DOI:** 10.3390/s22010191

**Published:** 2021-12-28

**Authors:** Małgorzata Domino, Marta Borowska, Natalia Kozłowska, Łukasz Zdrojkowski, Tomasz Jasiński, Graham Smyth, Małgorzata Maśko

**Affiliations:** 1 Department of Large Animal Diseases and Clinic, Institute of Veterinary Medicine, Warsaw University of Life Sciences, 02-787 Warsaw, Poland; malgorzata_domino@sggw.edu.pl (M.D.); natalia_kozlowska@sggw.edu.pl (N.K.); tomasz_jasinski@sggw.edu.pl (T.J.); 2 Institute of Biomedical Engineering, Faculty of Mechanical Engineering, Białystok University of Technology, 15-351 Bialystok, Poland; m.borowska@pb.edu.pl; 3 Menzies Health Institute Queensland, Griffith University School of Medicine, Southport, QLD 4222, Australia; grahamcsmyth@gmail.com; 4 Department of Animal Breeding, Institute of Animal Science, Warsaw University of Life Sciences, 02-787 Warsaw, Poland

**Keywords:** surface temperature, texture analysis, color model, digital image processing, mare

## Abstract

Infrared thermography (IRT) was applied as a potentially useful tool in the detection of pregnancy in equids, especially native or wildlife. IRT measures heat emission from the body surface, which increases with the progression of pregnancy as blood flow and metabolic activity in the uterine and fetal tissues increase. Conventional IRT imaging is promising; however, with specific limitations considered, this study aimed to develop novel digital processing methods for thermal images of pregnant mares to detect pregnancy earlier with higher accuracy. In the current study, 40 mares were divided into non-pregnant and pregnant groups and imaged using IRT. Thermal images were transformed into four color models (RGB, YUV, YIQ, HSB) and 10 color components were separated. From each color component, features of image texture were obtained using Histogram Statistics and Grey-Level Run-Length Matrix algorithms. The most informative color/feature combinations were selected for further investigation, and the accuracy of pregnancy detection was calculated. The image texture features in the RGB and YIQ color models reflecting increased heterogeneity of image texture seem to be applicable as potential indicators of pregnancy. Their application in IRT-based pregnancy detection in mares allows for earlier recognition of pregnant mares with higher accuracy than the conventional IRT imaging technique.

## 1. Introduction

Pregnancy detection is an important aspect of herd management, both in domestic and wild animals. In domestic animals, the most common methods of pregnancy detection are ultrasonography and hormone level measurements. However, both require direct contact with animals [1,2]. Such examinations may be stressful for an animal, or in the case of wild animals nearly impossible [3]. Thus, there is a need for the development of accurate, noninvasive, and contactless methods for pregnancy detection. Infrared thermography (IRT) is considered a valuable diagnostic method, which has previously been adapted to evaluate pregnancy in females of different species, such as zebras, rhinoceros, giraffes [4], pandas [5], cattle [6], and horses [7,8,9]. IRT measures the infrared spectrum of electromagnetic radiation emitted from the body surface [10]. This radiation represents the energy which is dissipated as heat [11], and is affected by a variety of factors, including internal factors such as local tissue metabolism and regional blood flow [12]. During pregnancy, intrauterine tissue proliferates, and uterine blood flow is increases. These physiological changes result in body surface temperature changes near the womb that are detectable by IRT [7].

IRT is considered an accurate imaging method, allowing for detection of even subtle changes in the body surface temperature before they can be detected by palpation [13]. However, some limitations should be considered when using IRT imaging in the detection of pregnancy. The surface body temperature gradients imaged in the abdominal area can be influenced by ambient temperature fluctuations [14,15], other weather conditions [10,16], and individual properties of horses’ body surface [17,18,19]. A high level of repeatability can be achieved and maintained in thermal images using standardized conditions of image acquisition [20,21]; however, the length of time it takes to detect pregnancy related surface temperature changes [7,8] and the accuracy of pregnancy detection [9] limit the application of IRT to the late stage of pregnancy. Therefore, this work focused on developing a methodology for the detection of pregnancy in mares by building a multifactorial approach for digital thermal image processing. This involved combining conventional IRT image acquisition and segmentation with advanced transformation of color models and extraction of image texture. Conventional IRT imaging is a promising modality, and with specific limitations considered, we aim to develop a novel method in the field of digital image processing (DIP).

DIP has been applied in the fields of material mechanics [22,23,24] and human medicine [25,26,27,28,29,30], and provides quantified, objective data considered to be more informative than conventional temperature measures. In materials science, DIP increased the efficiency of non-destructive materials testing, in the detection of damage to composite materials [22], aerospace structures [23], and building elements [24]. Moreover, in medical science, DIP increased the sensitivity and specificity of non-invasive diagnostic imaging, in the diagnosis of diabetes [25], skin cancer [26], and breast tumors [27]. However, the application of DIP to thermal images in equine medicine is only in the early stages of development [18,31]. In this study, two aspects of DIP, transformation to color models and image texture extraction, have been applied using IRT to enable earlier and more accurate detection of pregnancy in mares.

Thermal images are colorful, where the emitted infrared radiation is presented as the color gradient corresponding to the distribution of surface temperatures [15]. Conversely, image texture approaches require grayscale images as input [32,33,34,35,36]. Therefore, conversion of the IRT images to selected color components is required to transform the input to grayscale [36,37,38]. Certain mathematical functions are used to convert the color coordinates of light into three color components in three-dimensional space. Based on the position of the light color coordinates in space, the color models are determined [39,40]. In this research, Red/Green/Blue (RGB) color cube [39,41], Brightness/U-component/V-component (YUV) color spaces [39], Brightness/I-component/Q-component (YIQ) color spaces [39], and Hue/Saturation/Brightness (HSB) color coin [40] were the color models considered. Individual components of each color model may be used as an input for image texture analysis using the first order histogram [33] or the second order histogram [32] of the image intensity distribution to determine the pixel relations in Histogram Statistics (HS) and Gray Level Co-occurrence Matrix (GLCM) approaches, respectively. GLCM and Grey-Level Run-Length Matrix (GRLM), are both detailed approaches of Gray-Level Matrices (GLM). The GLM has been recently applied in equine medicine to detect the influence of a rider’s body weight on the thermography of the thoracolumbar region [31]. Moreover, GLM has successfully been used in human medicine to improve the extraction of texture features of ultrasound images [30], radiographic images [42], magnetic resonance images [29,43], and thermal images [27,44,45,46,47,48]. GLM describes a group of texture operators that map image function, image complexity, and statistics of pixel distribution [28] and has been reported to provide complex descriptions of image texture [28,29,43,44].

DIP applications have returned numerous component combinations of color models and features of image texture that have potential to be used in the detection of equine pregnancy; however, they must be evaluated in more detail prior to being used in the field. Therefore, this study aimed to transform thermal images obtained from non-pregnant and pregnant mares into four color models, separate ten color components, and extract features of image texture for each component. The applicability of color/feature combinations obtained were evaluated using specific criteria to select the most informative combinations. Finally, for the selected combinations, the accuracy of pregnancy detection was calculated for consecutive months.

## 2. Materials and Methods

### 2.1. Mares

The research was conducted in the Polish state stud farm Dobrzyniewo engaged in conservative breeding of horses of the Polish Konik breed. Out of a herd of 90 Konik Polski horses, 40 Konik Polski mares were selected to carry out IRT imaging. All studied mares were housed under the same conditions in all-day open stables. They were fed twice daily with an individualized portion of hay to maintain a healthy condition and had over 12 h of daily access to a large grassy pasture.

Mares were divided based on the ultrasonographic examination of the reproductive tract into one of two distinct groups of mares: non-pregnant and pregnant. The non-pregnant group was composed of 14 non-lactating mares (*n* = 14; age 5.47 ± 3.90 years; height 143.10 ± 2.09 cm), whereas the pregnant group was composed of 26 non-lactating mares (*n* = 26; age 6.28 ± 4.04 years; height 142.40 ± 2.12 cm). The pregnant group’s inclusion criteria were mares that had naturally mated in February and/or March and had a confirmed ultrasonographical pregnancy screened at 14 and 35-days post-ovulation, according to McCue’s protocol [2]. The non-pregnant group’s inclusion criteria were lack of mating during the current reproductive season and two negative results of ultrasonographical pregnancy examination. Ultrasonographic examination of the reproductive tract was conducted using an ultrasound scanner (MyLabOne, ESAOTE, Florence, Italy) and a linear 5 MHz transducer (ESAOTE, Florence, Italy). The experimental protocol was approved by the II Local Ethical Committee on Animal Testing in Warsaw on behalf of the National Ethical Committees on Animal Testing (No WAW2/007/2020, day 15 January 2020).

### 2.2. Data Collection

For all studied mares, the left lateral surface of the abdomen was imaged using a non-contact thermal camera (FLIR Therma CAM E60, FLIR Systems Brazil, Sorocaba, Brazil) with 0.99 emissivity and a temperature range from 10.0 to 40.0 °C. The impact of external conditions [14,15] was minimized by imaging mares in a closed space, devoid of wind and sun radiation. Thermal images were taken four times every two months, such that 26 mares enrolled in the study were divided into two subgroups basing on the age of pregnancy, where each subgroup concerned 13 mares. The first subgroup (*n* = 13) was imaged in the 4th, 6th, 8th, and 10th month of pregnancy, whereas the second (*n* = 13) in the 5th, 7th, 9th, and 11th month of pregnancy. Data collection began in April and was conducted until the last foaling took place in January, with the ambient temperature and humidity ranging from 1.0 °C and 50% to 24 °C and 90%, respectively. Each of a total of 160 thermal images were taken by the same researcher after a standardized preparation of the imaging area including brushing off dirt and mud 15 min before imaging [15]. The thermal camera was always set at the same distance (2.0 m from the imaged area) and positioned consistently (half of the vertical line through the tuber coxae). Thermal images obtained were then conventionally processed. The thermal image processing steps for conventional analysis of IRT measures included (i) image acquisition, (ii) segmentation of regions of interests (ROIs), and (iii) extraction of thermal features (the maximal temperature, the average temperature, the minimal temperature, and the area of the maximal temperature). Results from conventionally processed thermal images were previously documented [8,9].

### 2.3. Data Processing

**Figure 1:**
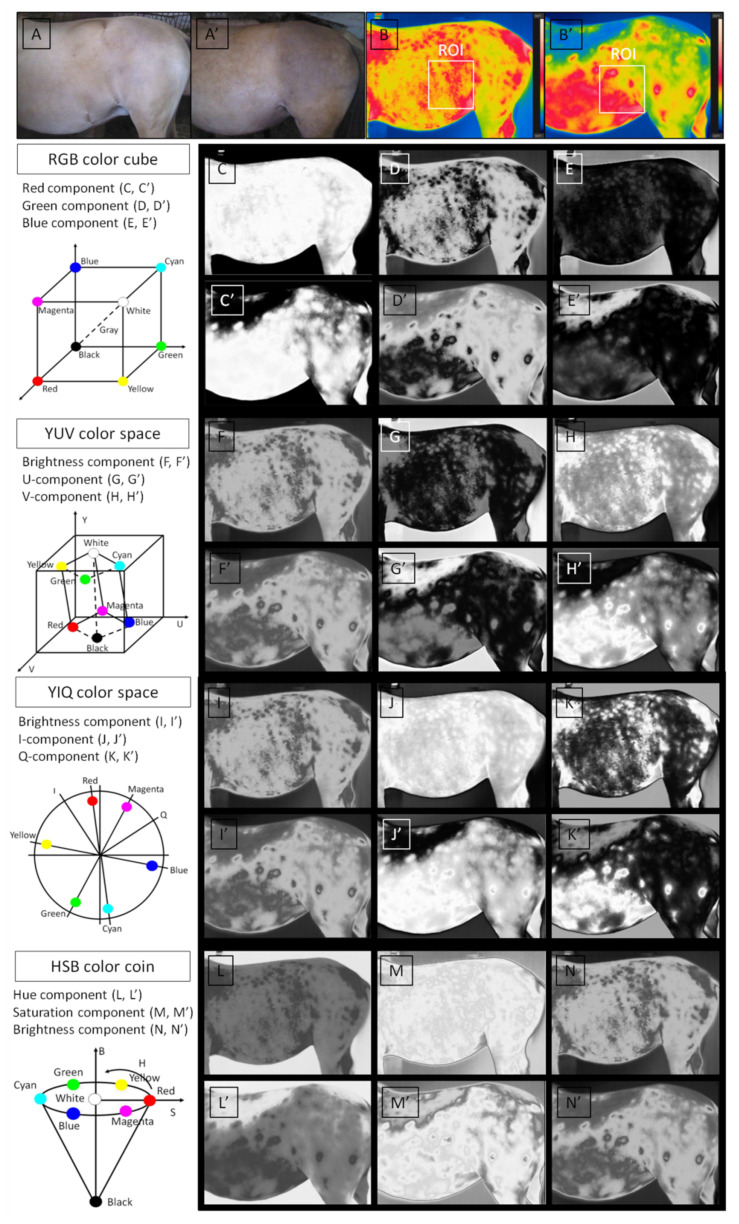
Thermal image processing for the analysis of image texture. Image acquisition, white light (**A**,**A’**) and thermal image (**B**,**B’**); segmentation of the region of interest (ROI) (**B**,**B’**); transformation to color models (**C**–**N’**): RGB color model (**C**–**E’**), YUV color model (**F**–**H’**), YIQ color model (**I**–**K’**), and HSB color model (**L**–**N’**). Subfigures (**A**–**N**) and (**A’**–**N’**) represent non-pregnant and pregnant mares, respectively.

The thermal image processing steps for image texture analysis included (i) image acquisition, (ii) segmentation of ROIs, (iii) transformation to color models, and (iv) extraction of image texture features using three analytical approaches: Histogram Statistics (HS), symmetric Gray Level Co-occurrence Matrix (GLCM), and asymmetric Gray Level Co-occurrence Matrix (GLCH). The first two steps were the same for the conventional analysis and the texture analysis of thermal images. All ROIs were manually annotated in the flank area. Each ROI was limited by the vertical line behind the tuber coxae, the dorsal edge of the abdomen, the caudal edge of the last rib, and the lower 2/3 of the abdomen height (Figure 1B,B’). The third and fourth steps were those of digital image processing.

#### 2.3.1. Transformation to Color Models

Grayscale images are required as input for the extraction of image texture features using QMazda Software. Therefore, the third step, transformation to color models, was conducted by QMazda Software as a way of image transformation to grayscale by conversion to selected color components [36,37,38]. IRT is based on the physical phenomenon, that all objects, including the equine body, with a temperature above absolute zero (−273 K) emit infrared (IR) radiation from their surface [49]. The radiated power is proportional to the fourth power of the object’s absolute temperature. The thermal camera detected by the radiated power in the IR spectrum focused IR radiation onto a two-dimensional array and used it to calculate the temperature of the object [10]. The output of the thermal camera is expressed as a thermogram or thermal images which is an image color coded for temperature [50]. In the presented approach, color analysis included the image transformation from the basic RGB color model to YUV, YIQ, and HSB color models which reflected the two-dimensional temperature assignation of the lateral aspect of the mares’ abdomens (Figure 1C–N’).

From the RGB color model, three components, the Red (R), Green (G) and Blue (B) components were separated without any conversion [36,37,38]. In other color spaces, the chrominance components were separated by transforming RGB spaces following the formulas below.

From the YUV color model, three components (a) Brightness component (Y), (b) U-component (U), and (c) V-component (V) were converted as follows [37]:

(a) $\left(299R+587G+114B\right)/1000$

(b) $\left(886B–587G–299R+886\Theta \right)/1772$

(c) $\left(–114B–587G+701R+701\Theta \right)/1402$

From the YIQ color model, the Brightness component (Y) remained the same as in the YUV color model, and two other components, (a) I-component (I) and (b) Q-component (Q), were converted as follows [36]:

(a) $\left(–\;3213B–2744G+5957R+5958\Theta \right)/11916$

(b) $\left(–3111B–5226G+2115R+5226\Theta \right)/10452$

For the HSB color model, the Brightness component (B) remained the same as Y in the YUV and YIQ color models, and two other components, (a) Hue (H) and (b) Saturation (S) components, were converted as follows [37]:

(a) $\frac{\Theta }{2\pi }arg\left(\frac{886B-587G-299R}{886}+j\frac{-114B-587G-701R}{701}\right)$

(b) $0.937\sqrt{{\left(\frac{886B-587G-299R}{886}\right)}^{2}+{\left(\frac{-114B-587G-701R}{701}\right)}^{2}}$

#### 2.3.2. Extraction of Image Texture Features

The fourth step, extraction of image texture features, was conducted using three approaches in annotated ROIs using QMazda Software [34,35]. Texture features of thermal images were calculated independently for individual R, G, B, Y, U, V, I, Q, H, and S components. The following three approaches were applied:

(i) Histogram Statistics (HS) is a usual method of image intensity analysis, based on the first order histogram. HS does not consider the special dependence on the intensity distribution. Moreover, the first order histogram features describe the overall number of pixels having certain intensity but independent of their location in the image [33]. For image I with the dimensions N×M and range of intensity $K\in \left[0\ldots 2^{n}-1\right]$ (n is the number of bits per pixel), the normalized histogram H is defined as:
  $$H\left(k\right)=\frac{number\;of\;pixels\;with\;value\;k\in \left[0\ldots 255\right]}{total\;number\;of\;pixels\;NxM}$$
  The 13 features from normalized H are defined as [33]:
  $$Mean=\frac{\sum_{i=1}^{N}\sum_{j=1}^{M}I\left(i,j\right)}{NM}\;Variance=\frac{\sum_{i=1}^{N}\sum_{j=1}^{M}{\left(I\left(i,j\right)-Mean\right)}^{2}}{NM}\;Skewness=\frac{\sum_{i=1}^{N}\sum_{j=1}^{M}{\left(I\left(i,j\right)-Mean\right)}^{3}{\left(\sqrt{Variance}\right)}^{-3}}{NM}\;Kurtosis=\frac{\sum_{i=1}^{N}\sum_{j=1}^{M}\left\{{\left(I\left(i,j\right)-Mean\right)}^{4}{\left(\sqrt{Variance}\right)}^{-4}\right\}-3}{NM}\;Perc01=\min\left(K\right):\overset{K}{\underset{k=0}{\sum }}H\left(k\right)\geq 0.01\;Perc10=\min\left(K\right):\overset{K}{\underset{k=0}{\sum }}H\left(k\right)\geq 0.10\;Perc50=\min\left(K\right):\overset{K}{\underset{k=0}{\sum }}H\left(k\right)\geq 0.50\;Perc90=\min\left(K\right):\overset{K}{\underset{k=0}{\sum }}H\left(k\right)\geq 0.90\;Perc99=\min\left(K\right):\overset{K}{\underset{k=0}{\sum }}H\left(k\right)\geq 0.99\;Domn01=k:max\left(\overset{K}{\underset{k=0}{\sum }}H\left(k\right)\right)\;Domn10=k:max\left(\overset{K-r}{\underset{k=0}{\sum }}\left(H\left(k+r\right)-H\left(k\right)\right)\right)\;Maxm01=\frac{1}{NM}max\left(\overset{K}{\underset{k=0}{\sum }}H\left(k\right)\right)\;Maxm10=\frac{1}{NM}max\left(\overset{K-r}{\underset{k=0}{\sum }}\left(H\left(k+r\right)-H\left(k\right)\right)\right)$$
(ii) Gray Level Co-occurrence Matrix (GLCM, GLCH) is a current method of image intensity analysis, based on the second order histogram. GLCM considers the mutual spatial relationship between pairs of image pixels with specific intensity levels. The GLCM method uses the second-order histogram of the image intensity distribution and can be calculated in different directions (horizontal in this study, vertical, 45°, 135°) and at different distances of pixel pairs ($d\in \left[1,\ldots ,9\right],\;d=1\;in\;this\;study$) [32]. For image I with the dimensions N×M and range of intensity $k,l\in \left[0\ldots 255\right]$, the co-occurrence matrix is defined as:

$$CoM\left(k,l\right)=\sum_{i=1}^{N}\sum_{j=1}^{M}\left\{\begin{array}{c} 1:if\;I\left(i,j\right)=k\cap I\left(i+∆i,j+∆j\right)=l\\ 0:\;otherwise \end{array}\right.$$

where $\left(∆i,∆j\right)$ is the offset between the pixel of interest and its neighbor. The $CoM\left(k,l\right)$ can be symmetric (GLCM) or asymmetric (GLCH). The normalized co-occurrence matrix (probability of co-occurrence) is defined as:

$$p\left(k,l\right)=\frac{CoM\left(k,l\right)}{\sum_{k=1}^{K}\sum_{l=1}^{K}CoM\left(k,l\right)}$$

The 11 Haralick features from normalized CoM matrix are defined as [32]:

$$\mathrm{AngScMom}=\sum_{k=1}^{K}\sum_{l=1}^{K}p{\left(k,l\right)}^{2}\;\mathrm{Contrast}=\sum_{m=0}^{K-1}m^{2}\left\{\sum_{k=1}^{K}\sum_{l=1}^{K}\begin{array}{c} p\left(k,l\right)\\ \left|k-l\right|=m \end{array}\right\}\;\mathrm{Correlat}=\sum_{k=1}^{K}\sum_{l=1}^{K}\frac{kl\;p\left(k,l\right)-\mu_{k}\mu_{l}}{\sigma_{k}\sigma_{l}}\;\mathrm{SumOfSqs}=\sum_{k=1}^{K}\sum_{l=1}^{K}{\left(k-\mu_{k}\right)}^{2}p\left(k,l\right)\;\mathrm{InvDefMom}=\sum_{k=1}^{K}\sum_{l=1}^{K}\frac{1}{1+{\left(k-l\right)}^{2}}p\left(k,l\right)\;\mathrm{SumAverg}=\sum_{m=2}^{2K}m\left\{\sum_{k=1}^{K}\sum_{l=1}^{K}\begin{array}{c} p\left(k,l\right)\\ k+l=m \end{array}\right\}\;\mathrm{SumVarnc}=\sum_{m=2}^{2K}{\left(m-SumAverg\right)}^{2}\left\{\sum_{k=1}^{K}\sum_{l=1}^{K}\begin{array}{c} p\left(k,l\right)\\ k+l=m \end{array}\right\}\;\mathrm{SumEntrp}=-\sum_{m=2}^{2K}\left\{\sum_{k=1}^{K}\sum_{l=1}^{K}\begin{array}{c} p\left(k,l\right)\\ k+l=m \end{array}\right\}\log\left\{\sum_{k=1}^{K}\sum_{l=1}^{K}\begin{array}{c} p\left(k,l\right)\\ k+l=m \end{array}\right\}\;\mathrm{Entropy}=-\sum_{k=1}^{K}\sum_{l=1}^{K}p\left(k,l\right)\log p\left(k,l\right)\;\mathrm{DifVarnc}=\sum_{m=0}^{K-1}{\left(m-\sum_{o=0}^{K-1}o\left\{\sum_{k=1}^{K}\sum_{l=1}^{K}\begin{array}{c} p\left(k,l\right)\\ \left|k-l\right|=o \end{array}\right\}\right)}^{2}\left\{\sum_{k=1}^{K}\sum_{l=1}^{K}\begin{array}{c} p\left(k,l\right)\\ k-l=m \end{array}\right\}\;\mathrm{DifEntrp}=-\sum_{m=0}^{K-1}\left\{\sum_{k=1}^{K}\sum_{l=1}^{K}\begin{array}{c} p\left(k,l\right)\\ k-l=m \end{array}\right\}\log\left\{\sum_{k=1}^{K}\sum_{l=1}^{K}\begin{array}{c} p\left(k,l\right)\\ k-l=m \end{array}\right\}$$

where

$$\mu_{k}=\sum_{k=0}^{K}\sum_{l=0}^{K}kp\left(k,l\right)\;\mu_{l}=\sum_{k=0}^{K}\sum_{l=0}^{K}lp\left(k,l\right)\;\sigma_{k}=\sum_{k=0}^{K}\sum_{l=0}^{K}{\left(k-\mu_{k}\right)}^{2}p\left(k,l\right)\;\sigma_{l}=\sum_{k=0}^{K}\sum_{l=0}^{K}{\left(l-\mu_{l}\right)}^{2}p\left(k,l\right).$$

### 2.4. Statistical Analysis

Statistical analysis was performed using GraphPad Prism6 software (GraphPad Software Inc., San Diego, CA, USA). Data from 35 image texture features (13 HS features, 11 GLCM features, and 11 GLCH features) were presented as data series for each color component independently, where each horse represented one realization. The numerical data in Supplementary Tables S1–S3 (Supplementary Materials) were presented as mean ± standard deviation (SD), whereas the numerical data on Supplementary Figures S1–S10 were presented as mean + SD. Data series were tested independently for univariate distributions using a Shapiro–Wilk normality test. Data analysis was performed in the following three steps: (i) Testing the differences between data series obtained in consecutive months for the non-pregnant group; (ii) testing the differences between the pooled non-pregnant group and data series obtained in consecutive months for the pregnant group; (iii) calculating the accuracy of pregnancy detection for consecutive months of pregnancy.

**Figure 2:**
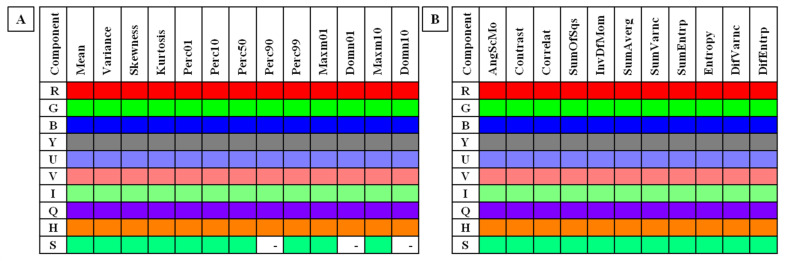
Features of (**A**) Histogram Statistics and (**B**) Gray Level Co-occurrence Matrix for each examined color component found not to be significantly different between months in the non-pregnant group. R—Red component in the RGB color model; G—Green component in the RGB color model; B—Blue component in the RGB color model; Y—Brightness component in the YUV/YIQ/HSB color models; U—U-component in the YUV color model; V—V-component in the YUV color model; I—I-component in the YIQ color model; Q—Q-component in the YIQ color model; H—Hue component in the HSB color model; S—Saturation component in the HSB color model. Skewness—skewness coefficient; Perc01, Perc10, Perc50, Perc90, Perc99—percentiles; Domn01, Domn10—dominants; Maxm01, Maxm10—maximum of moments; AngScMom—angular second moment/energy; Correlat—correlation; SumOfSqs—sum of squares; InvDefMom—inverse different moment/homogeneity; SumAverg—summation mean; SumVarnc—summation variance; SumEntrp—summation entropy; DifVarnc—differential variance; DifEntrp—differential entropy.

The comparisons between (i) data series for the non-pregnant group were assessed using the Ordinary one-way ANOVA followed by Tukey’s multiple comparisons test for Gaussian data and the Kruskal–Wallis test followed by the Dunn’s multiple comparisons test for non-Gaussian data. The alpha value was established as α = 0.05. When features do not differ significantly between consecutive months, the mean ± SD value was calculated as given in Supplementary Tables S1–S3. When features differed significantly, only the *p*-value was given. Only those features that did not differ between non-pregnant data series were summarized in Figure 2 and selected for further analysis.

**Figure 3:**
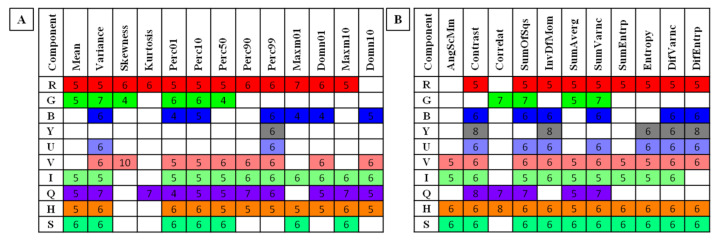
Features of (**A**) Histogram Statistics and (**B**) Gray Level Co-occurrence Matrix for each examined color component found to be significantly different between non-pregnant and pregnant groups from a given (numbered) month as compared to the end of pregnancy. R—Red component in the RGB color model; G—Green component in the RGB color model; B—Blue component in the RGB color model; Y—Brightness component in the YUV/YIQ/HSB color models; U—U-component in the YUV color model; V—V-component in the YUV color model; I—I-component in the YIQ color model; Q—Q-component in the YIQ color model; H—Hue component in the HSB color model; S—Saturation component in the HSB color model. Skewness—skewness coefficient; Perc01, Perc10, Perc50, Perc90, Perc99—percentiles; Domn01, Domn10—dominants; Maxm01, Maxm10—maximum of moments; AngScMom—angular second moment/energy; Correlat—correlation; SumOfSqs—sum of squares; InvDefMom—inverse different moment/homogeneity; SumAverg—summation mean; SumVarnc—summation variance; SumEntrp—summation entropy; DifVarnc—differential variance; DifEntrp—differential entropy.

**Figure 4:**
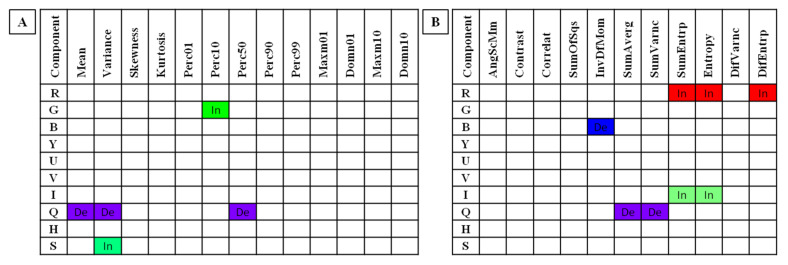
Features of (**A**) Histogram Statistics and (**B**) Gray Level Co-occurrence Matrix for each examined color component found to be significantly increasing (In) or decreasing (De) with the progression of pregnancy. R—Red component in the RGB color model; G—Green component in the RGB color model; B—Blue component in the RGB color model; Y—Brightness component in the YUV/YIQ/HSB color models; U—U-component in the YUV color model; V—V-component in the YUV color model; I—I-component in the YIQ color model; Q—Q-component in the YIQ color model; H—Hue component in the HSB color model; S—Saturation component in the HSB color model. Skewness—skewness coefficient; Perc01, Perc10, Perc50, Perc90, Perc99—percentiles; Domn01, Domn10—dominants; Maxm01, Maxm10—maximum of moments; AngScMom—angular second moment/energy; Correlat—correlation; SumOfSqs—sum of squares; InvDefMom—inverse different moment/homogeneity; SumAverg—summation mean; SumVarnc—summation variance; SumEntrp—summation entropy; DifVarnc—differential variance; DifEntrp—differential entropy.

The comparisons between (ii) data series for the non-pregnant and pregnant groups were assessed using the Ordinary one-way ANOVA followed by Tukey’s multiple comparisons test for Gaussian data and the Kruskal–Wallis test followed by the Dunn’s multiple comparisons test for non-Gaussian data. The alpha value was established as α = 0.05. When features differed significantly between the non-pregnant and pregnant groups between consecutive months, the month number was noted and presented in Supplementary Tables S4–S6. When the groups differed from a given month to the end of pregnancy, the feature was selected for visualization on a plot and summarized in Figure 3. On the plots in Supplementary Figures S1–S10 a comparison between non-pregnant and pregnant groups was visualized. Only those features that gradually increased or decreased within the pregnancy were summarized in Figure 4 and selected for further calculations.

The accuracy of (iii) pregnancy detection for consecutive months was calculated using three thresholds for gradually increasing features (mean, mean + SD, mean + 2SD) and for gradually decreasing features (mean, mean-SD, mean-2SD), respectively. The mare was annotated as pregnant (1) when the individual feature value was above threshold and annotated as non-pregnant (0) when below it. The same annotation was done in pregnant and non-pregnant groups. The sensitivity (Se), specificity (Sp), positive predictive value (PPV), and negative predictive value (NPV) of pregnancy detection were estimated. The values of Se, Sp, PPV, and NPV were calculated across the range of pregnancy proportions from 0.1 to 1.0 using standard formulae [51].

## 3. Results

Among 350 returned combinations of color components (*n* = 10) and image texture features (*n* = 35; HS *n* = 13, GLCM *n* = 11, GLCH *n* = 11), 127 combinations of HS, 110 combinations of GLCM, and 110 combinations of GLCH did not differ significantly between consecutive months of examination in the non-pregnant group. These combinations were summarized in Figure 2 and considered for further analysis after the first step of determining potential IRT indicators of pregnancy. For features which did not differ significantly between consecutive months, the mean ± SD value was calculated and presented as a pooled value of the non-pregnant group in Supplementary Tables S1–S3 available online. The pooled data series of 347 selected combinations were used for further comparisons with the pregnant groups.

Among 347 selected combinations of color components and image texture features, 77 combinations of HS, 76 combinations of GLCM, and 76 combinations of GLCH differed significantly between the non-pregnant and pregnant groups from a given month to the end of pregnancy. For the Red component in the RGB color model, 12 features of HS, nine features of GLCM, and nine features of GLCH were selected. For the Green component in the RGB color model, six features of HS, four features of GLCM, and four features of GLCH were selected. For the Blue component in the RGB color model, seven features of HS, six features of GLCM, and six features of GLCH were selected. For the Brightness component in the YUV/YIQ/HSB color models, one feature of HS, five features of GLCM, and five features of GLCH were selected. For the U-component in the YUV color model, two features of HS, seven features of GLCM, and seven features of GLCH were selected. For the V-component in the YUV color model, nine features of HS, 10 features of GLCM, and 10 features of GLCH were selected. For the I-component in the YIQ color model, 11 features of HS, nine features of GLCM, and nine features of GLCH were selected. For the Q-component in the YIQ color model, 11 features of HS, five features of GLCM, and five features of GLCH were selected. For the Hue component in the HSB color model, 11 features of HS, 11 features of GLCM, and 11 features of GLCH were selected. For the Saturation component in the HSB color model, seven features of HS, 10 features of GLCM, and 10 features of GLCH were selected. These combinations were summarized in Figure 3 and considered for further analysis after the second step of determining potential IRT indicators of pregnancy. Comparisons of image texture features were summarized in Supplementary Tables S4–S6 available online. No differences were found between the data tested for symmetric (GLCM) and asymmetric (GLCH) Gray Level Co-occurrence Matrices and therefore only the selection of features for GLCM was considered further. Among 229 selected combinations of color components and image texture features, GLCM feature reduction was found to be as equally useful as GLCH features, and therefore 153 combinations were further considered.

Among 153 selected combinations of color components and image texture features, five combinations of HS and eight combinations of GLCM increased or decreased significantly within consecutive months of pregnancy. With respect to the Red component in the RGB color model, no HS features were selected; however, three features of GLCM were selected (Figure S1 available online). The three features of GLCM were SumEntrp, Entropy, and DifEntrp which increased with progressing months of pregnancy. For the Green component in the RGB color model, one feature of HS was selected whereas no GLCM features were selected (Figure S2 available online). The GLCM feature selected was Perc10 which increased with progressing months of pregnancy. For the Blue component in the RGB color model, no HS features were selected and only one feature of GLCM was selected (Figure S3 available online). The GLCM featured selected was InvDefMom which decreased with progressing months of pregnancy. For the Brightness component in the YUV/YIQ/HSB color models, no features of either HS or GLCM were selected (Figure S4 available online). For both the U-component and the V-component in the YUV color model, no HS features were selected and one feature of GLCM was selected (Figures S5 and S6 available online). For the I-component in the YIQ color model, no HS features were selected; however, two GLCM features were selected (Figure S7 available online). The two selected GLCM features of the I-component were SumEntrp and Entropy which increased with progressing months of pregnancy. For the Q-component in the YIQ color model, three HS features and two GLCM features were selected (Figure S8 available online). These features were Mean, Variance, Perc50, SumAverg, and SumVarnc which all decreased with progressing months of pregnancy. For the Hue component in the HSB color model, no HS or GLCM features were selected (Figure S9 available online). For the Saturation component in the HSB color model, one HS feature was selected; however, no GLCM features were selected (Figure S10 available online). The one HS feature was Variance which increased with progressing months of pregnancy. These combinations are summarized in Figure 4 and were considered for further analysis after the third step of determining potential IRT indicators of pregnancy.

For all 13 selected combinations of color components and image texture features, the accuracy of pregnancy detection was summarized for the RGB color model (Table 1), YIQ color model (Table 2), and HSB color model (Table 3). The accuracy of pregnancy detection for the YUV color model was not calculated, due to a lack of selected combinations in this color model. In all three-color models, a salient observation is that the Se increased with higher threshold values (2SD > SD > mean) as well as with progressing months of pregnancy. For the first threshold (mean), Se ranged from 0.14 in the 4th and 5th months (SumEntrp of the Red component) to 1.00 in the 10th and 11th months (Perc10 of the Green component). For the second threshold (mean ± SD), Se ranged from 0.57 in the 4th month (Entropy of the Red component, SumEntrp of the I-component, Variance of the Q-component, and SumVarnc of the Q-component) to 1.00 in the 8th and 9th months (Mean of the Q-component) and then repetitively in 10th and 11th months (DifEntrp of the Red component, Perc10 of the Green component, Mean of the Q-component, Variance of the Q-component, SumVarnc of the Q-component, and SumVarnc of the Q-component). For the third threshold (mean ± 2SD), Se ranged from 0.86 in the 4th and 5th months (Perc10 of the Green component and Variance of the Q-component) and the 6th and 7th months (Perc10 of the Green component, to 1.00 in numerous months including the 4th month (SumEntrp of the Red component, InvDfMom of the Blue component, SumEntrp of the I-component, Entropy of the I-component, and Perc50 of the Q-component). For all considered thresholds, Sp varied within different months and features, therefore the applicability of potential IRT pregnancy indicators was considered and then discussed individually, during the fourth step of analysis.

## 4. Discussion

The first criterion, and thus the first step of determining potential IRT indicators of pregnancy was to determine which color/feature combinations were significantly different over time for mares in the non-pregnant group. It should be highlighted that homogeneity of data sets obtained from thermal images from the control group, in this research the non-pregnant group, is crucial for accurate group distinction [12,21,52,53]. Information obtained from thermal images of normal horses [18,20,54] provide a baseline of the expected values in the imaged area when comparing to the physiological [7,8,55] and pathological [13,56,57] changes observed in experimental groups. In the case of the abdominal area in mares, the maximal, average, and minimal temperatures on normal thermal images were recently reported [7,8,9] both for non-pregnant and pregnant mares. In previous research, the Gaussian distribution test was the only test used to explore the homogeneity of data. Uniform values would allow for easier diagnosis of pregnancy by increasing positive predictive value and decreasing negative predictive value [2,58,59]; the 350 combinations that passed this criterion were considered for further evaluation.

The second criterion, and thus the second step of determining potential IRT indicators of pregnancy was to determine which color/feature combinations differed significantly between the non-pregnant and pregnant groups from the first indicated month of pregnancy to the end of pregnancy. Bowers et al. [7] showed that IRT imaging can detect pregnancy in horses during late gestation, from the 9th month of pregnancy. Ultrasound allows the diagnosis of pregnancy on the 14th–16th day after ovulation [2]. One concern is that IRT may not be able to detect pregnancy as early as ultrasound and efforts were made therefore to determine how early pregnancy could be detected [8,9]. Using minimal temperature allowed the detection of pregnancy from the 8th month of gestation [8], whereas the maximal temperature, average temperature, and Area of Tmax from the 6th month [8,9]. The differences in thermal images were observed specifically in the smaller flank area of the lateral surface of the abdomen as opposed to the whole area of the lateral surface of the abdomen [8,9], probably due to the decreased amount of hair coat growth in the flank area [60] and thus lower local thermal insulation [19]. The flank area, therefore, was considered in this research. Of the cited features of thermal images, only the maximal temperature and the average temperature in the flank area [9] and the Area of Tmax [8] differed between examined groups from any given month to the end of pregnancy. Moreover, only the Area of Tmax increased as pregnancy progressed, and unlike measured maximal and average temperatures [9], it did not decrease as ambient temperature decreased [8]. It should be highlighted that body surface temperature is highly influenced by ambient temperature fluctuations [14,15], thus of the cited thermal features only one feature, the Area of Tmax, could pass the second criterion adopted in this study. We demonstrated as many as 153 combinations that met this criterion and thus were further evaluated as features that have an advantage over the conventional thermal features examined so far.

The third criterion, and thus the third step in determining potential IRT indicators of pregnancy, was to determine which feature/color combinations increase or decrease within the study period. The gradual increase or decrease of an indicator variable may allow the prediction of the time to parturition and evaluate the development of a free-living herd. In the case of wild horses, the ability to identify a pregnancy based on thermal imaging is limited by the lack of knowledge about mating [61], which can be improved by selecting continuously increasing features from the first indicated month until parturition. Such features would prove to be of high applicability using the proposed method, especially in wildlife research [3,61]. Of the cited features of thermal images, the Area of Tmax did not differ in the pregnant mares from the 6th month of pregnancy to delivery [8] and thus it could not pass the third criterion adopted in this study. Of the 13 combinations examined here which passed this criterion, noteworthy combinations were selected GLCM features in the RGB color model and features selected from HS and GLCM in the YIQ color model.

In different color models, the light color coordinates are mathematically converted into color components in three-dimensional space [40] representing the digitalized contained colors [39], therefore it was hypothesized that different color models will convey differing features of image texture. In the current study, the most combinations were selected in the RGB color model (three features in the Red component, and one feature each in each of the Green and Blue components) and the YIQ color model (two features in the I-component and five features in the Q-component). It is worth noting that in both color models, the same GLCM features were selected. SumEntrp and Entropy increased with the progression of pregnancy in both the Red and I-components. Recent research using the novel approach of thermal image analysis of the equine thoracolumbar region showed that both Entropy and DifEntrp were informative variables in differentiating between two physiological conditions: working under a heavy or a light rider [31]. The change in physiological condition was suggested to increase the degree of thermal energy dissipation detectable as an increased entropy measure. The high values of Entropy and SumEntrp indicate a high heterogeneity of texture [62] and may be a detectible feature in the progression of pregnancy. This result is in line with the successive increase of blood flow in the abdominal area, overall metabolic activity, and the proliferation of uterine and foetal tissues that require the pregnant mare to expend a large amount of energy [1,7,63]. This additional metabolic energy usage in pregnant mares leads to increases in heat emission from the body surface changing the conventional thermal pattern [7,8,9] but also the thermal image texture, as reported here.

While the selection of three features in the Red component is no surprise, the selection of five features in the Q-component may shed new light on the digital image processing of thermal images. In thermal images, high temperature is red-annotated and low temperature is blue-annotated [8,10,18]. Therefore, increased heterogeneity of texture in the Red component and decreased homogeneity indicated by the inverse different moment in the Blue component are consistent with conventional thermal results [7,8,9]. It was shown that the count of red-annotated pixels on thermal images, indicated by Area of Tmax, increased with the progression of pregnancy [8]. On the other hand, the Q-component corresponds to magenta-green axis [39] in the YIQ color model, which is sensitive to changes in luminance rather than hue or saturation changes [41]. With conventional thermal images, the medium-high temperature is magenta-annotated and the medium-low temperature is green-annotated [8,10,18] thus a decrease in the medium range of energy may also be a distinguishing feature of the progression of pregnancy. One might observe the described change in the Q-component when comparing Figure 1K,K’, representing non-pregnant and pregnant mares, respectively. In summary, we suspect that the increase in the count and mutual relations between extreme high red-annotated pixels as well as the decrease in the count and mutual relations between medium-high magenta-annotated pixels and extreme low blue-annotated pixels on the thermal images of the flank area may be potential IRT indicators of pregnancy.

Finally, the accuracy of pregnancy detection for consecutive months of pregnancy and selected color/feature combinations were then evaluated. In a recent study, the highest sensitivity and specificity of pregnancy detection using IRT was noted using the minimal temperature indicator in the 8th month of pregnancy [9]. Earlier pregnancy detection was reported with lower sensitivity and specificity using the average temperature indicator or with the lowest specificity using the maximal temperature indicator [9]. In the current study, with respect to the third threshold, comparable accuracy values were noted partially in the 4th and more clearly in the 5th month of pregnancy. In 6th month and certainly in 8th month of pregnancy the sensitivity and specificity of pregnancy detection in mares were much higher based on the texture features than conventional thermal measures. One may conclude, the application of DIP proposed here allows for earlier and more accurate detection of pregnancy as compared to conventional IRT imaging.

## 5. Conclusions

Thermal images obtained from non-pregnant and pregnant mares may be successfully transformed into four color models and their individual color components may be separated. Image texture features may then be successfully calculated from color components creating numerous color/feature combinations. Out of these combinations, the IRT image texture features representing the increase in extreme high red-annotated pixels, the decrease in medium-high magenta-annotated pixels, and extreme low blue-annotated pixels seem to be useful indicators of pregnancy. Application of these methods in IRT-based pregnancy detection allows for earlier recognition of pregnancy in mares with higher accuracy than conventional IRT imaging techniques.

## Figures and Tables

**Table 1:** Three thresholds (mean; mean ±SD (m ± SD); mean ±2SD (m ± 2SD)) used to estimate the sensitivity (Se), specificity (Sp), positive predictive value (PPV), and negative predictive value (NPV) of pregnancy detection using the selected features of Histogram Statistics and Gray Level Co-occurrence Matrix in the RGB color model.

Month	4th			5th			6th			7th			8th			9th			10th			11th		
Threshold	Mean	m ± SD	m ± 2SD	Mean	m ± SD	m ± 2SD	Mean	m ± SD	m ± 2SD	Mean	m ± SD	m ± 2SD	Mean	m ± SD	m ± 2SD	Mean	m ± SD	m ± 2SD	Mean	m ± SD	m ± 2SD	Mean	m ± SD	m ± 2SD
SumEntrp of the Red component																								
Se	0.14	0.64	1.00	0.14	0.64	1.00	0.43	0.71	1.00	0.50	0.71	1.00	0.50	0.86	1.00	0.50	0.86	1.00	0.79	0.93	1.00	0.86	0.93	1.00
Sp	1.00	1.00	0.54	1.00	1.00	1.00	1.00	1.00	1.00	1.00	1.00	1.00	1.00	1.00	1.00	1.00	1.00	1.00	1.00	1.00	1.00	1.00	1.00	1.00
PPV	1.00	1.00	0.70	1.00	1.00	1.00	1.00	1.00	1.00	1.00	1.00	1.00	1.00	1.00	1.00	1.00	1.00	1.00	1.00	1.00	1.00	1.00	1.00	1.00
NPV	0.52	0.72	1.00	0.52	0.72	1.00	0.62	0.76	1.00	0.65	0.76	1.00	0.65	0.87	1.00	0.65	0.87	1.00	0.81	0.93	1.00	0.87	0.93	1.00
Entropy of the Red component																								
Se	0.29	0.57	0.93	0.29	0.57	0.93	0.50	0.71	1.00	0.50	0.71	1.00	0.50	0.86	1.00	0.50	0.86	1.00	0.86	0.93	1.00	0.86	0.93	1.00
Sp	1.00	1.00	0.31	1.00	1.00	1.00	1.00	1.00	1.00	1.00	1.00	1.00	1.00	1.00	1.00	1.00	1.00	1.00	1.00	1.00	1.00	1.00	1.00	1.00
PPV	1.00	1.00	0.59	1.00	1.00	1.00	1.00	1.00	1.00	1.00	1.00	1.00	1.00	1.00	1.00	1.00	1.00	1.00	1.00	1.00	1.00	1.00	1.00	1.00
NPV	0.57	0.68	0.80	0.57	0.68	0.93	0.65	0.76	1.00	0.65	0.76	1.00	0.65	0.87	1.00	0.65	0.87	1.00	0.87	0.93	1.00	0.87	0.93	1.00
DifEntrp of the Red component																								
Se	0.36	0.71	0.93	0.36	0.71	0.93	0.43	0.79	0.93	0.50	0.79	0.93	0.50	0.86	1.00	0.50	0.86	1.00	0.71	1.00	1.00	0.79	1.00	1.00
Sp	1.00	0.77	0.23	1.00	1.00	1.00	1.00	1.00	1.00	1.00	1.00	1.00	1.00	1.00	1.00	1.00	1.00	1.00	1.00	1.00	1.00	1.00	1.00	1.00
PPV	1.00	0.77	0.57	1.00	1.00	1.00	1.00	1.00	1.00	1.00	1.00	1.00	1.00	1.00	1.00	1.00	1.00	1.00	1.00	1.00	1.00	1.00	1.00	1.00
NPV	0.59	0.71	0.75	0.59	0.76	0.93	0.62	0.81	0.93	0.65	0.81	0.93	0.65	0.87	1.00	0.65	0.87	1.00	0.76	1.00	1.00	0.81	1.00	1.00
Perc10 of the Green component																								
Se	0.64	0.79	0.86	0.64	0.79	0.86	0.64	0.86	0.86	0.64	0.86	0.86	0.79	0.93	1.00	0.79	0.93	1.00	1.00	1.00	1.00	1.00	1.00	1.00
Sp	0.69	0.46	0.31	1.00	0.46	0.23	0.92	0.77	0.54	1.00	0.92	0.92	1.00	0.92	0.85	1.00	0.92	0.85	1.00	1.00	1.00	1.00	1.00	1.00
PPV	0.69	0.61	0.57	1.00	0.61	0.55	0.90	0.80	0.67	1.00	0.92	0.92	1.00	0.93	0.88	1.00	0.93	0.88	1.00	1.00	1.00	1.00	1.00	1.00
NPV	0.64	0.67	0.67	0.72	0.67	0.60	0.71	0.83	0.78	0.72	0.86	0.86	0.81	0.92	1.00	0.81	0.92	1.00	1.00	1.00	1.00	1.00	1.00	1.00
InvDfMom of the Blue component																								
Se	0.50	0.79	1.00	0.50	0.79	1.00	0.43	0.71	0.93	0.43	0.71	0.93	0.57	0.86	1.00	0.57	0.86	1.00	0.50	0.93	1.00	0.50	0.93	1.00
Sp	0.15	0.00	0.00	0.54	0.46	0.31	0.85	0.69	0.69	0.77	0.69	0.54	0.92	0.92	0.54	1.00	1.00	1.00	1.00	0.85	0.85	0.85	0.85	0.69
PPV	0.39	0.46	0.52	0.54	0.61	0.61	0.75	0.71	0.76	0.67	0.71	0.68	0.89	0.92	0.70	1.00	1.00	1.00	1.00	0.87	0.88	0.78	0.87	0.78
NPV	0.22	0.00	1.00	0.50	0.67	1.00	0.58	0.69	0.90	0.56	0.69	0.88	0.67	0.86	1.00	0.68	0.87	1.00	0.65	0.92	1.00	0.61	0.92	1.00

SumEntrp—summation entropy; DifEntrp—differential entropy; Perc10—percentile 10; InvDefMom—inverse different moment/homogeneity.

**Table 2:** Three thresholds (mean; mean ±SD (m ± SD); mean ± 2SD (m ± 2SD)) used to estimate the sensitivity (Se), specificity (Sp), positive predictive value (PPV), and negative predictive value (NPV) of pregnancy detection using the selected features of Histogram Statistics and Gray Level Co-occurrence Matrix in the YIQ color model.

Month	4th			5th			6th			7th			8th			9th			10th			11th		
Threshold	Mean	m ± SD	m ± 2SD	Mean	m ± SD	m ± 2SD	Mean	m ± SD	m ± 2SD	Mean	m ± SD	m ± 2SD	Mean	m ± SD	m ± 2SD	Mean	m ± SD	m ± 2SD	Mean	m ± SD	m ± 2SD	Mean	m ± SD	m ± 2SD
SumEntrp of the I-component																								
Se	0.43	0.57	1.00	0.50	0.57	1.00	0.50	0.71	1.00	0.57	0.71	1.00	0.43	0.86	1.00	0.43	0.86	1.00	0.79	0.93	1.00	0.86	0.93	1.00
Sp	1.00	0.92	0.23	1.00	1.00	0.92	1.00	1.00	1.00	1.00	1.00	1.00	1.00	1.00	1.00	1.00	1.00	1.00	1.00	1.00	1.00	1.00	1.00	1.00
PPV	1.00	0.89	0.57	1.00	1.00	0.93	1.00	1.00	1.00	1.00	1.00	1.00	1.00	1.00	1.00	1.00	1.00	1.00	1.00	1.00	1.00	1.00	1.00	1.00
NPV	0.62	0.67	1.00	0.65	0.68	1.00	0.65	0.76	1.00	0.68	0.76	1.00	0.62	0.87	1.00	0.62	0.87	1.00	0.81	0.93	1.00	0.87	0.93	1.00
Entropy of the I-component																								
Se	0.29	0.64	1.00	0.29	0.64	1.00	0.50	0.64	1.00	0.50	0.71	1.00	0.57	0.79	1.00	0.57	0.86	1.00	0.79	0.93	1.00	0.79	0.93	1.00
Sp	1.00	0.85	0.23	1.00	1.00	0.85	1.00	1.00	1.00	1.00	1.00	1.00	1.00	1.00	1.00	1.00	1.00	1.00	1.00	1.00	1.00	1.00	1.00	1.00
PPV	1.00	0.82	0.58	1.00	1.00	0.88	1.00	1.00	1.00	1.00	1.00	1.00	1.00	1.00	1.00	1.00	1.00	1.00	1.00	1.00	1.00	1.00	1.00	1.00
NPV	0.57	0.69	1.00	0.57	0.72	1.00	0.65	0.72	1.00	0.65	0.76	1.00	0.68	0.81	1.00	0.68	0.87	1.00	0.81	0.93	1.00	0.81	0.93	1.00
Mean of the Q-component																								
Se	0.43	0.93	0.93	0.43	0.93	0.93	0.50	0.93	0.93	0.50	0.93	0.93	0.50	1.00	1.00	0.50	1.00	1.00	0.71	1.00	1.00	0.71	1.00	1.00
Sp	0.85	0.31	0.31	1.00	0.23	0.23	1.00	0.62	0.62	1.00	0.85	0.85	1.00	0.92	0.92	1.00	0.85	0.85	1.00	1.00	1.00	1.00	1.00	1.00
PPV	0.75	0.59	0.59	1.00	0.57	0.57	1.00	0.72	0.72	1.00	0.87	0.87	1.00	0.93	0.93	1.00	0.88	0.88	1.00	1.00	1.00	1.00	1.00	1.00
NPV	0.58	0.80	0.80	0.62	0.75	0.75	0.65	0.89	0.89	0.65	0.92	0.92	0.65	1.00	1.00	0.65	1.00	1.00	0.76	1.00	1.00	0.76	1.00	1.00
Variance of the Q-component																								
Se	0.36	0.57	0.86	0.36	0.57	0.86	0.36	0.79	0.93	0.36	0.79	0.93	0.64	0.93	1.00	0.64	0.93	1.00	0.79	1.00	1.00	0.79	1.00	1.00
Sp	0.46	0.23	0.15	0.54	0.23	0.08	0.85	0.54	0.00	0.92	0.85	0.38	0.92	0.85	0.15	0.85	0.85	0.23	1.00	0.92	0.54	1.00	1.00	0.69
PPV	0.42	0.44	0.52	0.45	0.44	0.50	0.71	0.65	0.50	0.83	0.85	0.62	0.90	0.87	0.56	0.82	0.87	0.58	1.00	0.93	0.70	1.00	1.00	0.78
NPV	0.40	0.33	0.50	0.44	0.33	0.33	0.55	0.70	1.00	0.57	0.79	0.83	0.71	0.92	1.00	0.69	0.92	1.00	0.81	1.00	1.00	0.81	1.00	1.00
Perc50 of the Q-component																								
Se	0.29	0.64	1.00	0.29	0.64	1.00	0.43	0.79	1.00	0.43	0.79	1.00	0.50	0.86	1.00	0.50	0.86	1.00	0.71	0.86	1.00	0.71	0.86	1.00
Sp	0.77	0.69	0.00	1.00	1.00	0.08	1.00	1.00	0.23	1.00	1.00	0.38	1.00	1.00	0.54	1.00	1.00	0.54	1.00	1.00	0.77	1.00	1.00	0.92
PPV	0.57	0.69	0.52	1.00	1.00	0.54	1.00	1.00	0.58	1.00	1.00	0.64	1.00	1.00	0.70	1.00	1.00	0.70	1.00	1.00	0.82	1.00	1.00	0.93
NPV	0.50	0.64	1.00	0.57	0.72	1.00	0.62	0.81	1.00	0.62	0.81	1.00	0.65	0.87	1.00	0.65	0.87	1.00	0.76	0.87	1.00	0.76	0.87	1.00
SumAverg of the Q-component																								
Se	0.43	0.64	0.93	0.43	0.64	0.93	0.43	0.71	0.93	0.50	0.79	0.93	0.50	0.86	1.00	0.50	0.86	1.00	0.71	0.86	1.00	0.71	0.86	1.00
Sp	0.85	0.69	0.31	1.00	1.00	0.23	1.00	1.00	0.62	1.00	1.00	0.85	1.00	1.00	0.92	1.00	1.00	0.85	1.00	1.00	1.00	1.00	1.00	1.00
PPV	0.75	0.69	0.59	1.00	1.00	0.57	1.00	1.00	0.72	1.00	1.00	0.87	1.00	1.00	0.93	1.00	1.00	0.88	1.00	1.00	1.00	1.00	1.00	1.00
NPV	0.58	0.64	0.80	0.62	0.72	0.75	0.62	0.76	0.89	0.65	0.81	0.92	0.65	0.87	1.00	0.65	0.87	1.00	0.76	0.87	1.00	0.76	0.87	1.00
SumVarnc of the Q-component																								
Se	0.36	0.57	0.92	0.36	0.57	0.92	0.79	0.79	0.92	0.79	0.79	0.92	0.64	0.86	0.92	0.64	0.93	1.00	0.36	1.00	1.00	0.36	1.00	1.00
Sp	0.46	0.23	0.15	0.54	0.23	0.08	0.85	0.54	0.00	0.92	0.92	0.38	0.92	0.85	0.15	0.92	0.85	0.23	1.00	0.92	0.54	1.00	1.00	0.69
PPV	0.42	0.44	0.52	0.45	0.44	0.50	0.85	0.65	0.48	0.92	0.92	0.60	0.90	0.86	0.52	0.90	0.87	0.57	1.00	0.93	0.68	1.00	1.00	0.76
NPV	0.40	0.33	0.67	0.44	0.33	0.50	0.79	0.70	0.00	0.80	0.80	0.83	0.71	0.85	0.67	0.71	0.92	1.00	0.59	1.00	1.00	0.59	1.00	1.00

SumEntrp—summation entropy; DifEntrp—differential entropy; Perc50—percentile 50; SumAverg—summation mean; SumVarnc—summation variance.

**Table 3:** Three thresholds (mean; mean ± SD (m ± SD); mean ±2SD (m ± 2SD)) used to estimate the sensitivity (Se), specificity (Sp), positive predictive value (PPV), and negative predictive value (NPV) of advanced pregnancy detection using the selected features of Histogram Statistics and Gray Level Co-occurrence Matrix in the HSB color model.

Month	4th			5th			6th			7th			8th			9th			10th			11th		
Threshold	Mean	m +SD	m +2SD	Mean	m +SD	m +2SD	Mean	m +SD	m +2SD	Mean	m +SD	m +2SD	Mean	m +SD	m +2SD	Mean	m +SD	m +2SD	Mean	m +SD	m +2SD	Mean	m +SD	m +2SD
Variance of the Saturation component																								
Se	0.71	0.86	0.93	0.71	0.86	0.93	0.57	0.86	0.93	0.57	0.86	0.93	0.71	0.93	0.93	0.71	0.93	0.93	0.86	1.00	1.00	0.86	1.00	1.00
Sp	0.38	0.38	0.31	0.85	0.77	0.62	1.00	1.00	1.00	1.00	1.00	1.00	1.00	1.00	1.00	1.00	1.00	1.00	1.00	1.00	1.00	1.00	1.00	1.00
PPV	0.56	0.60	0.59	0.83	0.80	0.72	1.00	1.00	1.00	1.00	1.00	1.00	1.00	1.00	1.00	1.00	1.00	1.00	1.00	1.00	1.00	1.00	1.00	1.00
NPV	0.56	0.71	0.80	0.73	0.83	0.89	0.68	0.87	0.93	0.68	0.87	0.93	0.76	0.93	0.93	0.76	0.93	0.93	0.87	1.00	1.00	0.87	1.00	1.00

## Data Availability

The data presented in this study are available on request from the corresponding author.

## Supplementary Materials

The following are available online at https://www.mdpi.com/article/10.3390/s22010191/s1, Figure S1: Twenty-one selected features (mean + SD) of the Histogram Statistics and Gray Level Co-occurrence Matrix for the Red component in the RGB color model compared between the non-pregnant group and pregnant group over consecutive months of pregnancy, Figure S2: Ten selected features (mean + SD) of the Histogram Statistics and Gray Level Co-occurrence Matrix for the Green component in the RGB color model compared between the non-pregnant group and pregnant group over consecutive months of pregnancy, Figure S3: Thirteen selected features (mean + SD) of the Histogram Statistics and Gray Level Co-occurrence Matrix for the Blue component in the RGB color model compared between the non-pregnant group and pregnant group over consecutive months of pregnancy, Figure S4: Six selected features (mean + SD) of the Histogram Statistics and Gray Level Co-occurrence Matrix for the Brightness component in the YUV/YIQ/HSB color models compared between the non-pregnant group and consecutive pregnant group over consecutive months of pregnancy, Figure S5: Nine selected features (mean + SD) of the Histogram Statistics and Gray Level Co-occurrence Matrix for the U-component in the YUV color model compared between the non-pregnant group and consecutive pregnant group over consecutive months of pregnancy, Figure S6: Nineteen selected features (mean + SD) of the Histogram Statistics and Gray Level Co-occurrence Matrix for the V-component in the YUV color model compared between the non-pregnant group and consecutive pregnant group over consecutive months of pregnancy, Figure S7: Twenty selected features (mean + SD) of the Histogram Statistics and Gray Level Co-occurrence Matrix for the I-component in the YIQ color model compared between the non-pregnant group and consecutive pregnant group over consecutive months of pregnancy, Figure S8: Sixteen selected features (mean + SD) of the Histogram Statistics and Gray Level Co-occurrence Matrix for the Q-component in the YIQ color model compared between the non-pregnant group and consecutive pregnant group over consecutive months of pregnancy, Figure S9: Twenty two selected features (mean + SD) of the Histogram Statistics and Gray Level Co-occurrence Matrix for the Hue component in the HSB color model compared between the non-pregnant group and consecutive pregnant group over consecutive months of pregnancy, Figure S10: Seventeen selected features (mean + SD) of the Histogram Statistics and Gray Level Co-occurrence Matrix for the Saturation component in the HSB color model compared between the non-pregnant group and consecutive pregnant group over consecutive months of pregnancy, Table S1: Features of Histogram Statistics (HS) for each examined color component in the non-pregnant group. When features did not differ significantly (*p* > 0.05) between consecutive months, the mean ±SD value was calculated as shown in the table, Table S2: Features of the symmetric Gray Level Co-occurrence Matrix (GLCM) for each examined color component in the non-pregnant group, Table S3: Features of the asymmetric Gray Level Co-occurrence Matrix (GLCH) for each examined color component in the non-pregnant group, Table S4: Comparison of Histogram Statistics (HS) features for each examined color component between the non-pregnant and pregnant groups, Table S5: Comparison of the symmetric Gray Level Co-occurrence Matrix (GLCM) features for each examined color component between the non-pregnant and pregnant groups, Table S6: Comparison of the asymmetric Gray Level Co-occurrence Matrix (GLCH) features for each examined color component between the non-pregnant and pregnant groups.
