# Supplementary material for: Advances in Thermal Image Analysis for the Detection of Pregnancy in Horses Using Infrared Thermography
Source: Sensors (Basel). 2021 Dec 28;22(1):191. doi: 10.3390/s22010191 (PMC8749616; doi:10.3390/s22010191)
Supplement: Supplementary file 1 [file sensors-22-00191-s001.zip › sensors-1495752-supplementary.pdf]

Supplementary Data for

Advances in thermal image analysis for the detection of pregnancy in horses using infrared thermography

Małgorzata Domino[1], Marta Borowska[2], Natalia Kozłowska[1], Łukasz Zdrojkowski[1*], Tomasz Jasiński[1], Graham Smyth[3], Małgorzata Maśko[4*]

[1] Department of Large Animal Diseases and Clinic, Institute of Veterinary Medicine, Warsaw University of Life Sciences, Warsaw, Poland; malgorzata_domino@sggw.edu.pl; natalia_kozlowska@sggw.edu.pl; tomasz_jasinski@sggw.edu.pl;

[2] Institute of Biomedical Engineering, Faculty of Mechanical Engineering, Białystok University of Technology, Bialystok, 15-351, Poland; m.borowska@pb.edu.pl

[3] Menzies Health Institute Queensland and Griffith University School of Medicine, Southport, QLD, 4222,Australia; grahamcsmyth@gmail.com

[4] Department of Animal Breeding, Institute of Animal Science, Warsaw University of Life Sciences, Warsaw, Poland;

* Correspondence: lukasz_zdrojkowski@sggw.edu.pl (Ł.Z.); malgorzata_masko@sggw.edu.pl (M.M.)

Table S1. Features of Histogram Statistics (HS) for each examined color component in the non-pregnant group. When features did not differ significantly (p>0.05) between consecutive months, the mean ±SD value was calculated as shown in the table. When features differed significantly (p<0.05), only p-values were given.

Component		Mean	Variance	Skewness	Kurtosis	Perc01	Perc10	Perc50	Perc90	Perc99	Maxm01	Domn01	Maxm10	Domn10
**R**	mean	245.5	149.9	3.061	18.04	203.5	234.5	248.9	252.0	253.0	0.204	251.7	0.889	237.0
	±SD	±3.4	±215.4	±1.811	±30.30	±33.9	±11.3	±1.4	±0.2	±0.1	±0.061	±1.4	±0.081	±0.1
	p	0.470	0.802	0.372	0.227	0.714	0.518	0.850	0.792	0.392	0.533	0.411	0.563	0.423
**G**	mean	117.0	3771	0.014	-1.128	25.79	36.64	116.7	198.7	210.4	0.039	108.3	0.249	98.20
	±SD	±24.0	±851	±0.530	±0.479	±7.93	±20.22	±40.3	±12.4	±4.4	±0.017	±93.9	±0.071	±85.36
	p	0.332	0.194	0.318	0.695	0.743	0.473	0.245	0.341	0.559	0.974	0.604	0.341	0.559
**B**	mean	36.07	554.2	-0.407	-0.616	2.107	7.518	33.82	67.86	87.54	0.040	19.70	0.344	17.25
	±SD	±11.08	±267.9	±0.381	±0.596	±2.612	±4.744	±14.33	±16.39	±18.92	±0.021	±25.91	±0.088	±23.17
	p	0.404	0.310	0.201	0.127	0.533	0.475	0.400	0.283	0.643	0.239	0.324	0.279	0.460
**Y**	mean	146.3	1069	0.110	-1.119	96.25	102.4	147.6	188.3	192.6	0.058	142.3	0.368	135.1
	±SD	±12.5	±239	±0.549	±0.581	±4.43	±11.4	±21.4	±4.29	±1.1	±0.021	±48.6	±0.093	±41.6
	p	0.342	0.158	0.308	0.520	0.220	0.390	0.252	0.250	0.150	0.503	0.451	0.900	0.368
**U**	mean	65.06	947.6	-0.118	-1.093	20.77	25.39	64.45	105.7	112.9	0.062	62.54	0.381	53.91
	±SD	±13.01	±253.6	±0.560	±0.565	±1.51	±4.49	±22.02	±12.5	±6.2	±0.027	±46.13	±0.111	±39.18
	p	0.319	0.193	0.341	0.491	0.144	0.387	0.370	0.246	0.230	0.372	0.317	0.912	0.102
**V**	mean	198.6	723.3	0.326	-0.809	142.3	161.4	201.1	231.5	235.4	0.052	225.0	0.354	207.4
	±SD	±10.4	±205.6	±0.430	±0.615	±15.8	±10.8	±15.2	±7.0	±2.6	±0.027	±24.2	±0.096	±23.3
	p	0.274	0.391	0.388	0.516	0.709	0.469	0.233	0.232	0.247	0.469	0.619	0.258	0.856
**I**	mean	213.9	156.9	0.827	0.864	180.6	197.2	215.8	227.6	229.2	0.083	226.4	0.577	210.5
	±SD	±4.5	±85.7	±0.563	±3.714	±17.0	±7.4	±5.0	±2.0	±0.7	±0.031	±6.2	±0.113	±5.3
	p	0.327	0.494	0.823	0.770	0.684	0.451	0.243	0.192	0.396	0.290	0.180	0.416	0.765
**Q**	mean	29.4	1457	0.065	-1.105	65.64	77.54	131.1	178.1	184.4	0.049	159.6	0.303	136.8
	±SD	±15.5	±354	±0.509	±0.424	±6.21	±10.14	±25.6	±12.7	±5.10	±0.026	±47.0	±0.093	±47.6
	p	0.321	0.423	0.311	0.639	0.651	0.407	0.333	0.513	0.093	0.268	0.389	0.198	0.969
**H**	mean	92.72	267.9	-0.105	-1.002	69.16	72.20	91.82	114.4	122.4	0.091	80.32	0.483	81.89
	±SD	±6.72	±77.1	±0.510	±0.639	±2.45	±5.46	±11.12	±4.5	±8.0	±0.053	±19.33	±0.091	±16.82
	p	0.306	0.278	0.440	0.577	0.260	0.192	0.326	0.470	0.623	0.382	0.785	0.386	0.240
**S**	mean	194.7	97.03	1.106	3.455	167.1	183.5	196.0	-	205.0	206.8	-	201.4	-
	±SD	±3.4	±91.46	±0.922	±5.831	±18.1	±8.4	±2.8		±0.700	±0.437		±7.2	
	p	0.363	0.955	0.349	0.190	0.849	0.747	0.320	0.033	0.338	0.628	0.301	0.816	0.015

R - Red component in the RGB color model; G - Green component in the RGB color model; B - Blue component in the RGB color model; Y - Brightness component in the YUV/YIQ/HSB color models; U - U-component in the YUV color model; V - V-component in the YUV color model; I - I-component in the YIQ color model; Q - Q-component in the YIQ color model; H - Hue component in the HSB color model; S - Saturation component in the HSB color model. Skewness - skewness coefficient; Perc01, Perc10, Perc50, Perc90, Perc99 - percentiles; Domn01, Domn10 - dominants; Maxm01, Maxm10 - maximum of moments; p - the level of marginal significance; SD - standard deviation.

Table S2. Features of the symmetric Gray Level Co-occurrence Matrix (GLCM) for each examined color component in the non-pregnant group. When features did not differ significantly (p>0.05) between consecutive months, the mean ±SD value was calculated as shown in the table.

Component		AngScMom	Contrast	Correlat	SumOfSqs	InvDfMom	SumAverg	SumVarnc	SumEntrp	Entropy	DifVarnc	DifEntrp
**R**	mean	0.053	1.38 ±1.86	0.990	149.1	0.765	493.0	595.0	1.481	1.707	1.019	0.422
	±SD	±0.021		±0.007	±215.1	±0.046	±6.8	±859.4	±0.172	±0.227	±1.535	±0.078
	p	0.932	0.855	0.717	0.812	0.520	0.463	0.802	0.584	0.613	0.937	0.533
**G**	mean	0.003	22.59	0.997	3769	0.364	235.9	15055	2.456	3.255	12.50	0.979
	±SD	±0.002	±7.96	±0.001	±851	±0.052	±48.1	±3404	±0.061	±0.136	±4.25	±0.070
	p	0.931	0.938	0.522	0.199	0.920	0.333	0.197	0.996	0.989	0.940	0.998
**B**	mean	0.005	3.200	0.997	554.3	0.558	74.22	2214	2.155	2.642	1.657	0.632
	±SD	±0.003	±1.204	±0.002	±268.0	±0.059	±22.19	±1071	±0.109	±0.17	±0.598	±0.065
	p	0.117	0.941	0.389	0.310	0.680	0.404	0.310	0.108	0.258	0.989	0.885
**Y**	mean	0.006	7.374	0.996	1069	0.491	294.5	4269	2.163	2.752	4.151	0.770
	±SD	±0.003	±2.535	±0.001	±240	±0.053	±24.9	±959	±0.069	±0.137	±1.360	±0.065
	p	0.425	>0.999	0.370	0.162	0.868	0.343	0.164	0.902	0.887	0.965	0.996
**U**	mean	0.007	5.342	0.997	947.5	0.531	132.2	3785	2.148	2.674	3.023	0.709
	±SD	±0.004	±2.007	±0.001	±253.5	±0.060	±26.0	±1014	±0.087	±0.165	±1.066	±0.070
	p	0.539	0.928	0.295	0.187	0.973	0.319	0.187	0.626	0.887	0.819	0.989
**V**	mean	0.005	4.190	0.997	722.4	0.520	399.3	2886	2.178	2.716	2.174	0.680
	±SD	±0.003	±1.464	±0.001	±205.5	±0.408	±20.8	±822	±0.089	±0.114	±0.782	±0.053
	p	0.261	0.836	0.747	0.400	0.810	0.274	0.400	0.210	0.239	0.729	0.861
**I**	mean	0.014	1.297	0.995	156.5	0.676	429.8	624.8	1.851	2.170	0.728	0.476
	±SD	±0.005	±0.630	±0.002	±85.6	±0.033	±8.9	±342.0	±0.110	±0.129	±0.431	±0.045
	p	0.142	0.959	0.653	0.511	0.706	0.327	0.511	0.260	0.134	0.995	0.969
**Q**	mean	0.004	7.868	0.997	1456	0.471	260.9	5815	2.282	2.898	4.321	0.783
	±SD	±0.003	±2.838	±0.001	±353	±0.053	±31.1	±1414	±0.073	±0.130	±1.500	±0.068
	p	0.582	0.920	0.371	0.234	0.924	0.311	0.237	0.579	0.860	0.773	0.989
**H**	mean	0.016	1.510	0.997	267.8	0.682	187.4	1070	1.919	2.234	0.895	0.500
	±SD	±0.011	±0.541	±0.001	±77.1	±0.049	±13.5	±308	±0.091	±0.134	±0.296	±0.053
	p	0.324	0.880	0.389	0.291	0.885	0.306	0.291	0.540	0.735	0.764	0.921
**S**	mean	0.011	2.793	0.983	97.06	0.587	391.4	385.4	1.731	2.189	1.545	0.594
	±SD	±0.002	±1.280	±0.006	±91.58	±0.045	±6.8	±365.5	±0.105	±0.160	±0.778	±0.068
	p	0.884	0.932	0.959	0.950	0.947	0.366	0.946	0.823	0.822	0.874	0.861

R - Red component in the RGB color model; G - Green component in the RGB color model; B - Blue component in the RGB color model; Y - Brightness component in the YUV/YIQ/HSB color models; U - U-component in the YUV color model; V - V-component in the YUV color model; I - I-component in the YIQ color model; Q - Q-component in the YIQ color model; H - Hue component in the HSB color model; S - Saturation component in the HSB color model. AngScMom - angular second moment/energy; Correlat - correlation; SumOfSqs - sum of squares; InvDefMom - inverse different moment/homogeneity; SumAverg - summation mean; SumVarnc - summation variance; SumEntrp - summation entropy; DifVarnc - differential variance; DifEntrp - differential entropy; p - the level of marginal significance; SD - standard deviation.

Table S3. Features of the asymmetric Gray Level Co-occurrence Matrix (GLCH) for each examined color component in the non-pregnant group. When features did not differ significantly (p>0.05) between consecutive months, the mean ±SD value was calculated as shown in the table.

Component		AngScMom	Contrast	Correlat	SumOfSqs	InvDfMom	SumAverg	SumVarnc	SumEntrp	Entropy	DifVarnc	DifEntrp
**R**	mean	0.052	1.384	0.990	148.7	0.765	493.0	595.0	1.481	1.703	1.019	0.422
	±SD	±0.021	±1.861	±0.007	±215.5	±0.046	±6.8	±859.4	±0.172	±0.24	±1.535	±0.078
	p	0.823	0.855	0.748	0.810	0.520	0.463	0.802	0.584	0.608	0.937	0.533
**G**	mean	0.003	22.59	0.997	3766	0.364	235.9	15055	2.456	3.238	12.50	0.979
	±SD	±0.002	±7.96	±0.001	±851	±0.052	±48.1	±3404	±0.061	±0.136	±4.25	±0.070
	p	0.970	0.998	0.493	0.201	0.963	0.277	0.197	0.985	0.977	0.940	0.998
**B**	mean	0.005	3.200	0.997	554.0	0.558	74.22	2214	2.155	2.636	1.657	0.632
	±SD	±0.003	±1.204	±0.002	±267.8	±0.059	±22.19	±1071	±0.109	±0.176	±0.598	±0.065
	p	0.118	0.941	0.377	0.310	0.680	0.404	0.310	0.108	0.240	0.989	0.885
**Y**	mean	0.006	7.374	0.996	1069	0.491	294.5	4269	2.163	2.744	4.151	0.770
	±SD	±0.003	±2.535	±0.001	±239	±0.053	±24.9	±958	±0.070	±0.137	±1.360	±0.065
	p	0.435	>0.999	0.339	0.167	0.868	0.343	0.164	0.902	0.875	0.964	0.996
**U**	mean	0.007	5.342	0.997	946.8	0.531	132.2	3785	2.148	2.674	3.023	0.709
	±SD	±0.004	±2.007	±0.001	±253.3	±0.060	±26.0	±1014	±0.087	±0.165	±1.066	±0.070
	p	0.539	0.928	0.318	0.195	0.973	0.319	0.187	0.626	0.897	0.819	0.989
**V**	mean	0.005	4.190	0.997	721.4	0.520	399.3	2886	2.178	2.718	2.174	0.680
	±SD	±0.003	±1.464	±0.001	±205.7	±0.408	±20.8	±822	±0.089	±0.089	±0.782	±0.053
	p	0.257	0.836	0.721	0.426	0.810	0.274	0.400	0.210	0.224	0.729	0.861
**I**	mean	0.014	1.297	0.995	156.2	0.676	429.8	624.8	1.851	2.166	0.728	0.476
	±SD	±0.005	±0.630	±0.002	±85.7	±0.033	±8.9	±342.0	±0.110	±0.128	±0.431	±0.045
	p	0.134	0.959	0.656	0.527	0.706	0.327	0.511	0.260	0.130	0.995	0.969
**Q**	mean	0.004	7.868	0.997	1454	0.471	260.9	5815	2.282	2.889	4.321	0.783
	±SD	±0.003	±2.838	±0.001	±356	±0.053	±31.1	±1414	±0.073	±0.131	±1.500	±0.068
	p	0.587	0.920	0.350	0.266	0.924	0.311	0.237	0.579	0.855	0.773	0.989
**H**	mean	0.016	1.510	0.997	267.5	0.682	187.4	1070	1.919	2.229	0.895	0.500
	±SD	±0.011	±0.541	±0.001	±77.2	±0.049	±13.5	±308	±0.091	±0.135	±0.296	±0.053
	p	0.324	0.880	0.374	0.290	0.885	0.306	0.291	0.540	0.696	0.764	0.921
**S**	mean	0.011	2.793	0.983	97.13	0.587	391.4	385.4	1.731	2.189	1.545	0.594
	±SD	±0.003	±1.280	±0.006	±91.69	±0.045	±6.8	±365.5	±0.105	±0.160	±0.778	±0.068
	p	0.889	0.932	0.959	0.950	0.947	0.366	0.946	0.823	0.814	0.874	0.861

R - Red component in the RGB color model; G - Green component in the RGB color model; B - Blue component in the RGB color model; Y - Brightness component in the YUV/YIQ/HSB color models; U - U-component in the YUV color model; V - V-component in the YUV color model; I - I-component in the YIQ color model; Q - Q-component in the YIQ color model; H - Hue component in the HSB color model; S - Saturation component in the HSB color model. AngScMom - angular second moment/energy; Correlat - correlation; SumOfSqs - sum of squares; InvDefMom - inverse different moment/homogeneity; SumAverg - summation mean; SumVarnc - summation variance; SumEntrp - summation entropy; DifVarnc - differential variance; DifEntrp - differential entropy; p - the level of marginal significance; SD - standard deviation.

Table S4. Comparison of Histogram Statistics (HS) features for each examined color component between the non-pregnant and pregnant groups. When features differed significantly (p<0.05) between the non-pregnant group and pregnant group for consecutive months, the number of month (mo) was noted. When the groups differed from a given month to the end of pregnancy, the value was marked in bold.

Component		Mean	Variance	Skewness	Kurtosis	Perc01	Perc10	Perc50	Perc90	Perc99	Maxm01	Domn01	Maxm10	Domn10
**R**	NP/P diff.	**5-11 mo**	**5-11 mo**	**6-11 mo**	**6-11 mo**	**5-11 mo**	**5-11 mo**	**5-11 mo**	**6-11 mo**	**6-11 mo**	**7-11 mo**	**6-11 mo**	**5-11 mo**	6, 8-11 mo
	p	**<0.0001**	**<0.0001**	**<0.0001**	**<0.0001**	**<0.0001**	**<0.0001**	**<0.0001**	**<0.0001**	**<0.0001**	**<0.0001**	**<0.0001**	**<0.0001**	<0.0001
**G**	NP/P diff.	**5-11 mo**	**7-11 mo**	**4-11 mo**	6, 8-11 mo	**6-11 mo**	**6-11 mo**	**4-11 mo**	4, 5, 11 mo	4 mo	4 mo	4 mo	4, 5, 7-11 mo	4, 5 mo
	p	**<0.0001**	**<0.0001**	**<0.0001**	<0.0001	**<0.0001**	**<0.0001**	**<0.0001**	<0.0001	0.0009	0.007	0.002	<0.0001	0.013
**B**	NP/P diff.	6, 9, 10 mo	**6-11 mo**	5, 7-11 mo	5, 7, 8, 10, 11 mo	**4-11 mo**	**5-11 mo**	4, 5, 7, 8, 10, 11 mo	6, 7, 9-11 mo	**6-11 mo**	**4-11 mo**	**4-11 mo**	6, 8, 9, 11 mo	**5-11 mo**
	p	<0.0001	**<0.0001**	<0.0001	<0.0001	**<0.0001**	**<0.0001**	<0.0001	<0.0001	**<0.0001**	**<0.0001**	**<0.0001**	<0.0001	**<0.0001**
**Y**	NP/P diff.	4, 5 mo	5 mo	4, 5, 7, 8 mo	4, 5 ,7, 8 mo	6, 7, 9-11 mo	4, 5 mo	4, 5 mo	6 mo	**6-11 mo**	6, 9, 10 mo	-	4 mo	-
	p	0.0007	0.001	<0.0001	0.0001	<0.0001	0.0005	0.002	0.009	**<0.0001**	<0.0001	0.158	0.013	0.977
**U**	NP/P diff.	5 mo	**6-11 mo**	4, 5, 7-11 mo	4, 5, 7-11 mo	4 mo	4 mo	4, 5, 7, 8 mo	6, 7, 9-11 mo	**6-11 mo**	4 mo	-	4 mo	-
	p	0.0002	**<0.0001**	<0.0001	<0.0001	0.024	0.025	<0.0001	<0.0001	**<0.0001**	0.002	0.0750	0.007	0.427
**V**	NP/P diff.	**6-11 mo**	**10-11 mo**	6, 9-11 mo	4, 5 mo	**5-11 mo**	**5-11 mo**	**6-11 mo**	**6-11 mo**	**6-11 mo**	5, 7-11 mo	**6-11 mo**	5, 7-11 mo	**6-11 mo**
	p	**<0.0001**	**<0.0001**	<0.0001	0.0008	**<0.0001**	**<0.0001**	**<0.0001**	**<0.0001**	**<0.0001**	<0.0001	**<0.0001**	<0.0001	**<0.0001**
**I**	NP/P diff.	**5-11 mo**	**5-11 mo**	6 mo	9-10 mo	**5-11 mo**	**5-11 mo**	**5-11 mo**	**6-11 mo**	**6-11 mo**	**6-11 mo**	**6-11 mo**	**6-11 mo**	**6-11 mo**
	p	**<0.0001**	**<0.0001**	0.028	<0.0001	**<0.0001**	**<0.0001**	**<0.0001**	**<0.0001**	**<0.0001**	**<0.0001**	**<0.0001**	**<0.0001**	**<0.0001**
**Q**	NP/P diff.	**5-11 mo**	**7-11 mo**	5, 7-11 mo	**7-11 mo**	**4-11 mo**	**5-11 mo**	**5-11 mo**	**7-11 mo**	**6-11 mo**	-	**5-11 mo**	**7-11 mo**	**5-11 mo**
	p	**<0.0001**	**<0.0001**	<0.0001	**<0.0001**	**<0.0001**	**<0.0001**	**<0.0001**	**<0.0001**	**<0.0001**	0.1508	**<0.0001**	**<0.0001**	**<0.0001**
**H**	NP/P diff.	**5-11 mo**	**6-11 mo**	7, 8, 11 mo	4, 5, 7, 8, 11 mo	**6-11 mo**	**6-11 mo**	**5-11 mo**	**5-11 mo**	**5-11 mo**	**5-11 mo**	**5-11 mo**	**6-11 mo**	**5-11 mo**
	p	**<0.0001**	**<0.0001**	<0.0001	<0.0001	**<0.0001**	**<0.0001**	**<0.0001**	**<0.0001**	**<0.0001**	**<0.0001**	**<0.0001**	**<0.0001**	**<0.0001**
**S**	NP/P diff.	**6-11 mo**	**6-11 mo**	5 mo	10, 11 mo	**6-11 mo**	**6-11 mo**	**6-11 mo**	**-**	6, 8-11 mo	**6-11 mo**	-	**6-11 mo**	-
	p	**<0.0001**	**<0.0001**	0.0002	<0.0001	**<0.0001**	**<0.0001**	**<0.0001**	**-**	<0.0001	**<0.0001**	-	**<0.0001**	-

R – Red component in the RGB color model; G – Green component in the RGB color model; B – Blue component in the RGB color model; Y – Brightness component in the YUV/YIQ/HSB color models; U – U-component in the YUV color model; V – V-component in the YUV color model; I – I-component in the YIQ color model; Q – Q-component in the YIQ color model; H – Hue component in the HSB color model; S – Saturation component in the HSB color model. Skewness – skewness coefficient; Perc01, Perc10, Perc50, Perc90, Perc99 - percentiles; Domn01, Domn10 – dominants; Maxm01, Maxm10 – maximum of moments; p – the level of marginal significance; NP/P diff. – differences between non-pregnant and pregnant groups.

Table S5. Comparison of the symmetric Gray Level Co-occurrence Matrix (GLCM) features for each examined color component between the non-pregnant and pregnant groups. When features differed significantly (p<0.05) between the non-pregnant group and pregnant groups for consecutive months, the number of month (mo) was noted. When the groups differed from a given month to the end of pregnancy, the value was marked in bold.

**Component**		AngScMom	Contrast	Correlat	SumOfSqs	InvDfMom	SumAverg	SumVarnc	SumEntrp	Entropy	DifVarnc	DifEntrp
**R**	NP/P diff.	5, 7-11 mo	**5-11 mo**	4, 7 mo	**5-11 mo**	**5-11 mo**	**5-11 mo**	**5-11 mo**	**5-11 mo**	**5-11 mo**	**5-11 mo**	**5-11 mo**
	p	<0.0001	**<0.0001**	<0.0001	**<0.0001**	**<0.0001**	**<0.0001**	**<0.0001**	**<0.0001**	**<0.0001**	**<0.0001**	**<0.0001**
**G**	NP/P diff.	4 mo	8, 9 mo	**7-11 mo**	**7-11 mo**	4, 8, 9 mo	**5-11 mo**	**7-11 mo**	4, 7-11 mo	4 mo	8-10 mo	4, 8, 9 mo
	p	0.001	<0.0001	**<0.0001**	**<0.0001**	<0.0001	**<0.0001**	**<0.0001**	<0.0001	0.0007	<0.0001	<0.0001
**B**	NP/P diff.	4, 5, 11 mo	**6-11 mo**	5, 8-11 mo	**6-11 mo**	**6-11 mo**	6, 9, 10 mo	**6-11 mo**	6, 9 mo	6, 8-11 mo	**6-11 mo**	**6-11 mo**
	p	0.0004	**<0.0001**	<0.0001	**<0.0001**	**<0.0001**	<0.0001	**<0.0001**	<0.0001	<0.0001	**<0.0001**	**<0.0001**
**Y**	NP/P diff.	6, 8-11 mo	**8-11 mo**	5, 7-11 mo	5 mo	**8-11 mo**	4, 5 mo	5 mo	6, 9-11 mo	**6-11 mo**	**6-11 mo**	**8-11 mo**
	p	<0.0001	**<0.0001**	<0.0001	0.001	**<0.0001**	0.0006	0.002	<0.0001	**<0.0001**	**<0.0001**	**<0.0001**
**U**	NP/P diff.	4, 9, 10 mo	**6-11 mo**	5, 8-11 mo	**6-11 mo**	**6-11 mo**	5 mo	**6-11 mo**	6, 9-11 mo	**6-11 mo**	**6-11 mo**	**6-11 mo**
	p	<0.0001	**<0.0001**	<0.0001	**<0.0001**	**<0.0001**	0.0002	**<0.0001**	<0.0001	**<0.0001**	**<0.0001**	**<0.0001**
**V**	NP/P diff.	**5-11 mo**	**6-11 mo**	8-10 mo	**6-11 mo**	**6-11 mo**	**5-11 mo**	**6-11 mo**	**5-11 mo**	**5-11 mo**	**6-11 mo**	**6-11 mo**
	p	**<0.0001**	**<0.0001**	<0.0001	**<0.0001**	**<0.0001**	**<0.0001**	**<0.0001**	**<0.0001**	**<0.0001**	**<0.0001**	**<0.0001**
**I**	NP/P diff.	**5-11 mo**	**6-11 mo**	4 mo	**5-11 mo**	**6-11 mo**	**5-11 mo**	**5-11 mo**	**5-11 mo**	**5-11 mo**	**6-11 mo**	4, 6, 7 mo
	p	**<0.0001**	**<0.0001**	<0.0001	**<0.0001**	**<0.0001**	**<0.0001**	**<0.0001**	**<0.0001**	**<0.0001**	**<0.0001**	<0.0001
**Q**	NP/P diff.	-	**8-11 mo**	**7-11 mo**	**7-11 mo**	4, 8-10 mo	**5-11 mo**	**7-11 mo**	7, 8, 10, 11 mo	4 mo	8-10 mo	8, 9, 10 mo
	p	0.137	**<0.0001**	**<0.0001**	**<0.0001**	<0.0001	**<0.0001**	**<0.0001**	<0.0001	0.004	<0.0001	<0.0001
**H**	NP/P diff.	**6-11 mo**	**6-11 mo**	**8-11 mo**	**6-11 mo**	**6-11 mo**	**5-11 mo**	**6-11 mo**	**6-11 mo**	**6-11 mo**	**6-11 mo**	**6-11 mo**
	p	**<0.0001**	**<0.0001**	**<0.0001**	**<0.0001**	**<0.0001**	**<0.0001**	**<0.0001**	**<0.0001**	**<0.0001**	**<0.0001**	**<0.0001**
**S**	NP/P diff.	**6-11 mo**	**6-11 mo**	4, 9 mo	**6-11 mo**	**6-11 mo**	**6-11 mo**	**6-11 mo**	**6-11 mo**	**6-11 mo**	**6-11 mo**	**6-11 mo**
	p	**<0.0001**	**<0.0001**	0.0004	**<0.0001**	**<0.0001**	**<0.0001**	**<0.0001**	**<0.0001**	**<0.0001**	**<0.0001**	**<0.0001**

R – Red component in the RGB color model; G – Green component in the RGB color model; B – Blue component in the RGB color model; Y – Brightness component in the YUV/YIQ/HSB color models; U – U-component in the YUV color model; V – V-component in the YUV color model; I – I-component in the YIQ color model; Q – Q-component in the YIQ color model; H – Hue component in the HSB color model; S – Saturation component in the HSB color model. AngScMom – angular second moment/energy; Correlat – correlation; SumOfSqs – sum of squares; InvDefMom – inverse different moment/homogeneity; SumAverg – summation mean; SumVarnc – summation variance; SumEntrp – summation entropy; DifVarnc – differential variance; DifEntrp – differential entropy; p – the level of marginal significance; NP/P diff. – differences between non-pregnant and pregnant groups.

Table S6. Comparison of the asymmetric Gray Level Co-occurrence Matrix (GLCH) features for each examined color component between the non-pregnant and pregnant groups. When features differed significantly ($p<0.05$) between the non-pregnant group and pregnant groups for consecutive months, the number of month (mo) was noted. When the groups differed from a given month to the end of pregnancy, the value was marked in bold.

**Component**		AngScMom	Contrast	Correlat	SumOfSqs	InvDfMom	SumAverg	SumVarnc	SumEntrp	Entropy	DifVarnc	DifEntrp
**R**	NP/P diff.	5, 7-11 mo	**5-11 mo**	4, 7 mo	**5-11 mo**	**5-11 mo**	**5-11 mo**	**5-11 mo**	**5-11 mo**	**5-11 mo**	**5-11 mo**	**5-11 mo**
	p	<0.0001	**<0.0001**	<0.0001	**<0.0001**	**<0.0001**	**<0.0001**	**<0.0001**	**<0.0001**	**<0.0001**	**<0.0001**	**<0.0001**
**G**	NP/P diff.	4 mo	8, 9 mo	**7-11 mo**	**7-11 mo**	4, 8, 9 mo	**5-11 mo**	**7-11 mo**	4, 7-11 mo	4 mo	8-10 mo	4, 8, 9 mo
	p	0.001	<0.0001	**<0.0001**	**<0.0001**	<0.0001	**<0.0001**	**<0.0001**	<0.0001	0.0009	<0.0001	<0.0001
**B**	NP/P diff.	4, 5, 11 mo	**6-11 mo**	5, 8-11 mo	**6- 11 mo**	**6-11 mo**	6, 9, 10 mo	**6-11 mo**	6, 9 mo	6, 8-11 mo	**6-11 mo**	**6-11 mo**
	p	0.0005	**<0.0001**	<0.0001	**<0.0001**	**<0.0001**	<0.0001	**<0.0001**	<0.0001	<0.0001	**<0.0001**	**<0.0001**
**Y**	NP/P diff.	6, 8-11 mo	**8-11 mo**	5, 7-11 mo	5 mo	**8-11 mo**	4, 5 mo	5 mo	6, 9-11 mo	**6-11 mo**	**6-11 mo**	**8-11 mo**
	p	<0.0001	**<0.0001**	<0.0001	0.001	**<0.0001**	0.0006	0.002	<0.0001	**<0.0001**	**<0.0001**	**<0.0001**
**U**	NP/P diff.	4, 9, 10 mo	**6-11 mo**	5, 8-11 mo	**6-11 mo**	**6-11 mo**	5 mo	**6-11 mo**	6, 9-11 mo	**6-11 mo**	**6-11 mo**	**6-11 mo**
	p	<0.0001	**<0.0001**	<0.0001	**<0.0001**	**<0.0001**	0.0002	**<0.0001**	<0.0001	**<0.0001**	**<0.0001**	**<0.0001**
**V**	NP/P diff.	**5-11 mo**	**6-11 mo**	8-10 mo	**6-11 mo**	**6-11 mo**	**5-11 mo**	**6-11 mo**	**5-11 mo**	**5-11 mo**	**6-11 mo**	**6-11 mo**
	p	**<0.0001**	**<0.0001**	<0.0001	**<0.0001**	**<0.0001**	**<0.0001**	**<0.0001**	**<0.0001**	**<0.0001**	**<0.0001**	**<0.0001**
**I**	NP/P diff.	**5-11 mo**	**6-11 mo**	4 mo	**5-11 mo**	**6-11 mo**	**5-11 mo**	**5-11 mo**	**5-11 mo**	**5-11 mo**	**6-11 mo**	4, 6, 7 mo
	p	**<0.0001**	**<0.0001**	<0.0001	**<0.0001**	**<0.0001**	**<0.0001**	**<0.0001**	**<0.0001**	**<0.0001**	**<0.0001**	<0.0001
**Q**	NP/P diff.	-	**8-11 mo**	**7-11 mo**	**7-11 mo**	4, 8-10 mo	**5-11 mo**	**7-11 mo**	7, 8, 10, 11 mo	4 mo	8-10 mo	8, 9, 10 mo
	p	0.137	**<0.0001**	**<0.0001**	**<0.0001**	<0.0001	**<0.0001**	**<0.0001**	<0.0001	0.004	<0.0001	<0.0001
**H**	NP/P diff.	**6-11 mo**	**6-11 mo**	**8-11 mo**	**6-11 mo**	**6-11 mo**	**5-11 mo**	**6-11 mo**	**6-11 mo**	**6-11 mo**	**6-11 mo**	**6-11 mo**
	p	**<0.0001**	**<0.0001**	**<0.0001**	**<0.0001**	**<0.0001**	**<0.0001**	**<0.0001**	**<0.0001**	**<0.0001**	**<0.0001**	**<0.0001**
**S**	NP/P diff.	**6-11 mo**	**6-11 mo**	4, 9 mo	**6-11 mo**	**6-11 mo**	**6-11 mo**	**6-11 mo**	**6-11 mo**	**6-11 mo**	**6-11 mo**	**6-11 mo**
	p	**<0.0001**	**<0.0001**	0.0004	**<0.0001**	**<0.0001**	**<0.0001**	**<0.0001**	**<0.0001**	**<0.0001**	**<0.0001**	**<0.0001**

R – Red component in the RGB color model; G – Green component in the RGB color model; B – Blue component in the RGB color model; Y – Brightness component in the YUV/YIQ/HSB color models; U – U-component in the YUV color model; V – V-component in the YUV color model; I – I-component in the YIQ color model; Q – Q-component in the YIQ color model; H – Hue in the HSB component color model; S – Saturation component in the HSB color model. AngScMom – angular second moment/energy; Correlat – correlation; SumOfSqs – sum of squares; InvDefMom – inverse different moment/homogeneity; SumAverg – summation mean; SumVarnc – summation variance; SumEntrp – summation entropy; DifVarnc – differential variance; DifEntrp – differential entropy; p – the level of marginal significance; NP/P diff. – differences between non-pregnant and pregnant groups.

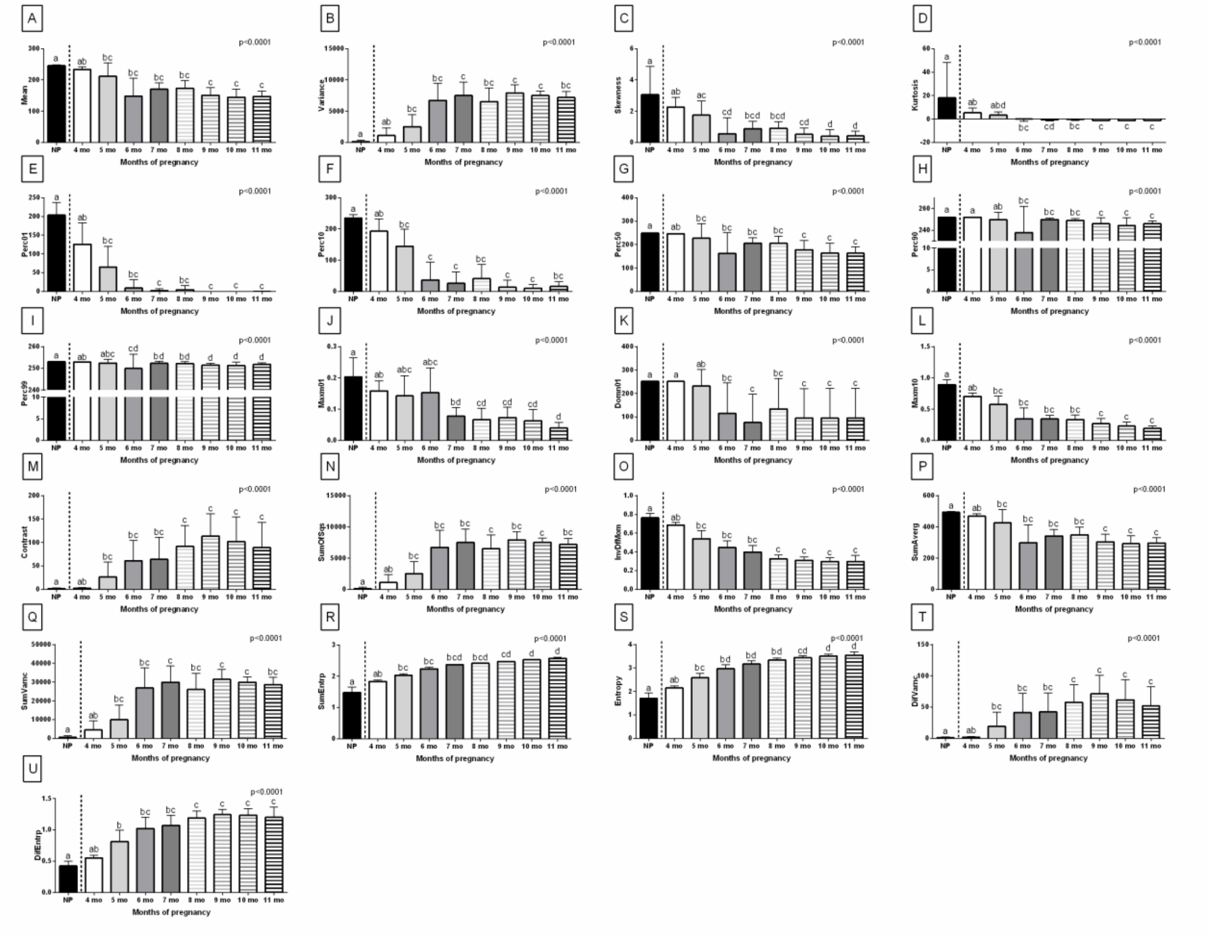

Figure S1. Twenty-one selected features (mean +SD) of the Histogram Statistics and Gray Level Co-occurrence Matrix for the Red component in the RGB color model compared between the non-pregnant group and pregnant group over consecutive months of pregnancy. Differences between groups were indicated with individual p-values when $p<0.05$. Different superscripts (a, b, c, d) were statistically different. Consecutive plots represent (**A**) Mean; (**B**) Variance; (**C**) Skewness - skewness coefficient; (**D**) Kurtosis; (**E**) Perc01 - percentile 01; (**F**) Perc10 - percentile 10; (**G**) Perc50 - percentile 50; (**H**) Perc90 - percentile 90; (**I**) Perc99 - percentile 99; (**J**) Maxm01 - maximum of moment 01; (**K**) Domn01 - dominant 01; (**L**) Maxm10 - maximum of moment 10; (**M**) Contrast; (**N**) SumOfSqs - sum of squares; (**O**) InvDefMom - inverse different moment/homogeneity; (**P**) SumAverg - summation mean; (**Q**) SumVarnc - summation variance; (**R**) SumEntrp - summation entropy; (**S**) Entropy; (**T**) DifVarnc - differential variance; (**U**) DifEntrp - differential entropy.

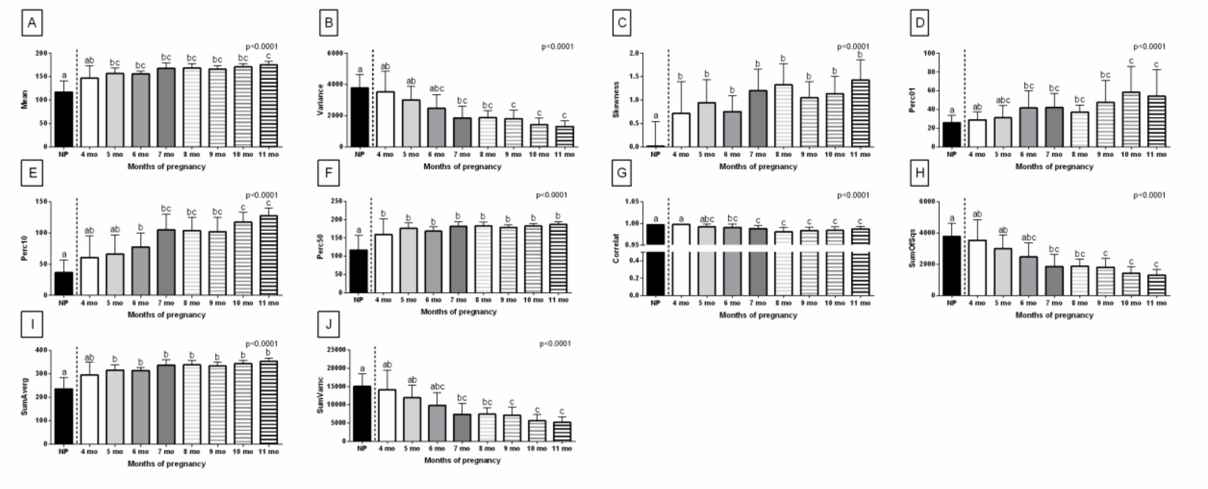

Figure S2. Ten selected features (mean +SD) of the Histogram Statistics and Gray Level Co-occurrence Matrix for the Green component in the RGB color model compared between the non-pregnant group and pregnant group over consecutive months of pregnancy. Differences between groups were indicated with individual p-value when p<0.05. Different superscripts (a, b, c) were statistically different. Consecutive plots represent (**A**) Mean; (**B**) Variance; (**C**) Skewness - skewness coefficient; (**D**) Perc01 - percentile 01; (**E**) Perc10 - percentile 10; (**F**) Perc50 - percentile 50; (**G**) Correlat - correlation; (**H**) SumOfSqs - sum of squares; (**I**) SumAverg - summation mean; (**J**) SumVarnc - summation variance.

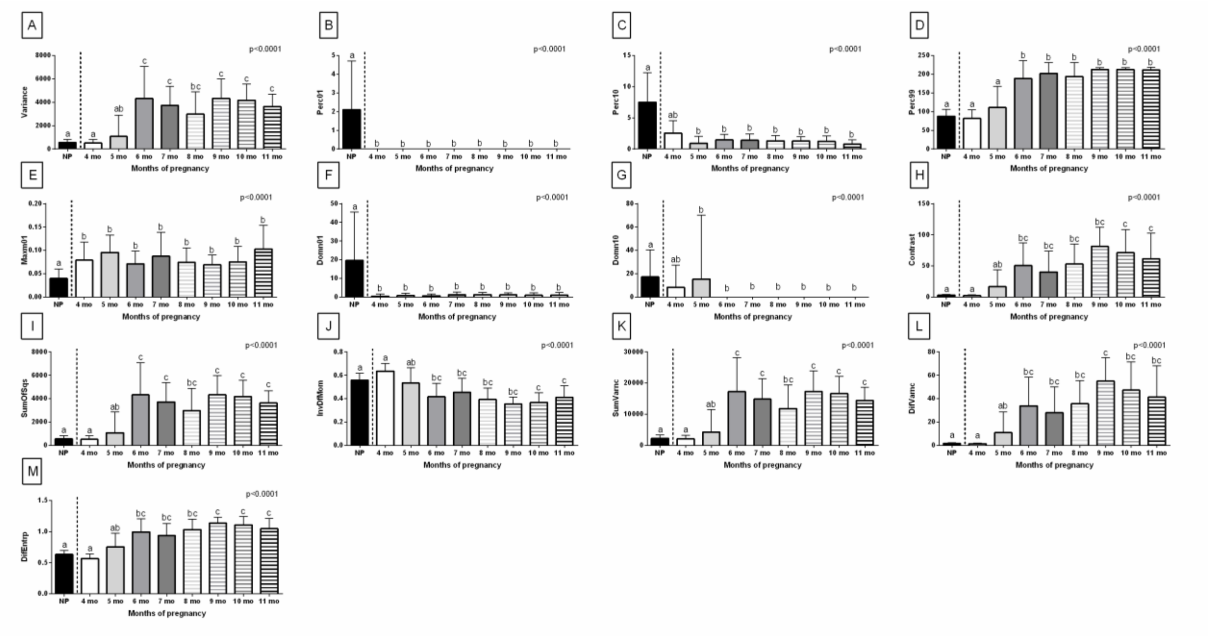

Figure S3. Thirteen selected features (mean +SD) of the Histogram Statistics and Gray Level Co-occurrence Matrix for the Blue component in the RGB color model compared between the non-pregnant group and pregnant group over consecutive months of pregnancy. Differences between groups were indicated with individual p-value when p<0.05. Different superscripts within the pregnant group (a, b, c) were statistically different. Consecutive plots represent (**A**) Variance; (**B**) Perc01 - percentile 01; (**C**) Perc10 - percentile 10; (**D**) Perc99 - percentile 99; (**E**) Maxm01 - maximum of moment 01; (**F**) Domn01 - dominant 01; (**G**) Domn10 - dominant 10; (**H**) Contrast; (**I**) SumOfSqs - sum of squares; (**J**) InvDefMom - inverse different moment/homogeneity; (**K**) SumVarnc - summation variance; (**L**) DifVarnc - differential variance; (**M**) DifEntrp - differential entropy.

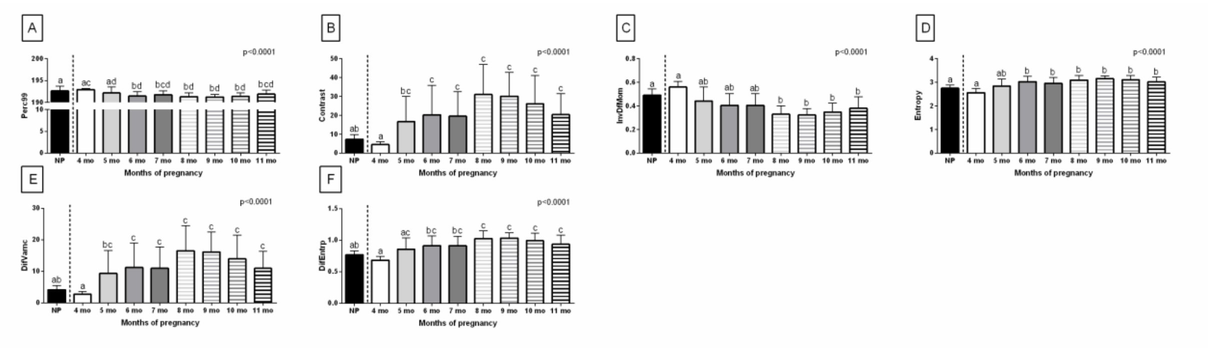

Figure S4. Six selected features (mean +SD) of the Histogram Statistics and Gray Level Co-occurrence Matrix for the Brightness component in the YUV/YIQ/HSB color models compared between the non-pregnant group and consecutive pregnant group over consecutive months of pregnancy. Differences between groups were indicated with individual p-value when p<0.05. Different superscripts within pregnant (a, b, c, d) were statistically different. Consecutive plots represent (**A**) Perc99 - percentile 99; (**B**) Contrast; (**C**) InvDefMom - inverse different moment/homogeneity; (**D**) Entropy; (**E**) DifVarnc - differential variance; (**F**) DifEntrp - differential entropy.

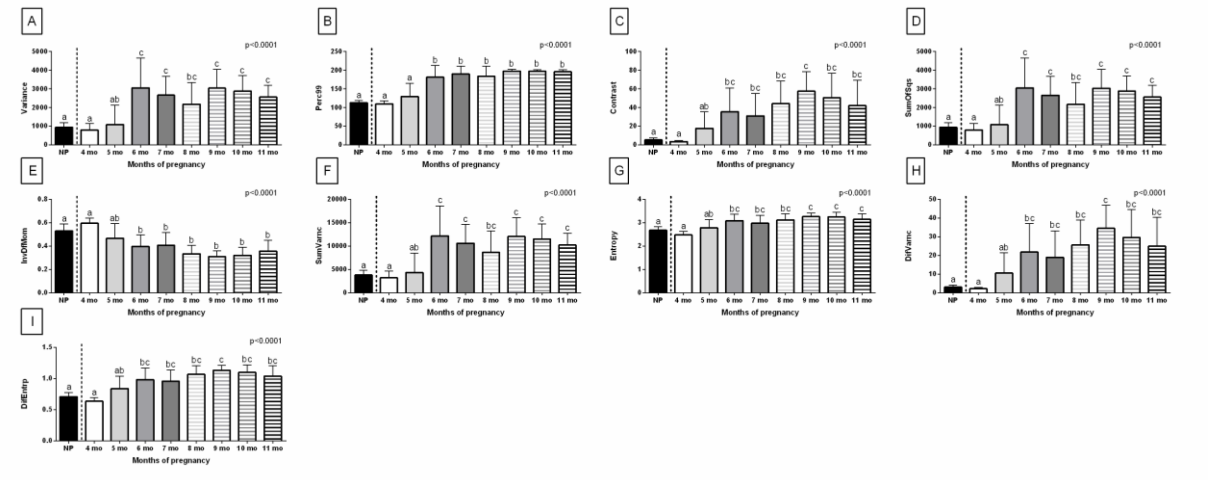

Figure S5. Nine selected features (mean +SD) of the Histogram Statistics and Gray Level Co-occurrence Matrix for the U-component in the YUV color model compared between the non-pregnant group and consecutive pregnant group over consecutive months of pregnancy. Differences between groups were indicated with individual p-value when p<0.05. Different superscripts within pregnant (a, b, c) were statistically different. Consecutive plots represent (**A**) Variance; (**B**) Perc99 - percentile 99; (**C**) Contrast; (**D**) SumOfSqs - sum of squares; (**E**) InvDefMom - inverse different moment/homogeneity; (**F**) SumVarnc - summation variance; (**G**) Entropy; (**H**) DifVarnc - differential variance; (**I**) DifEntrp - differential entropy.

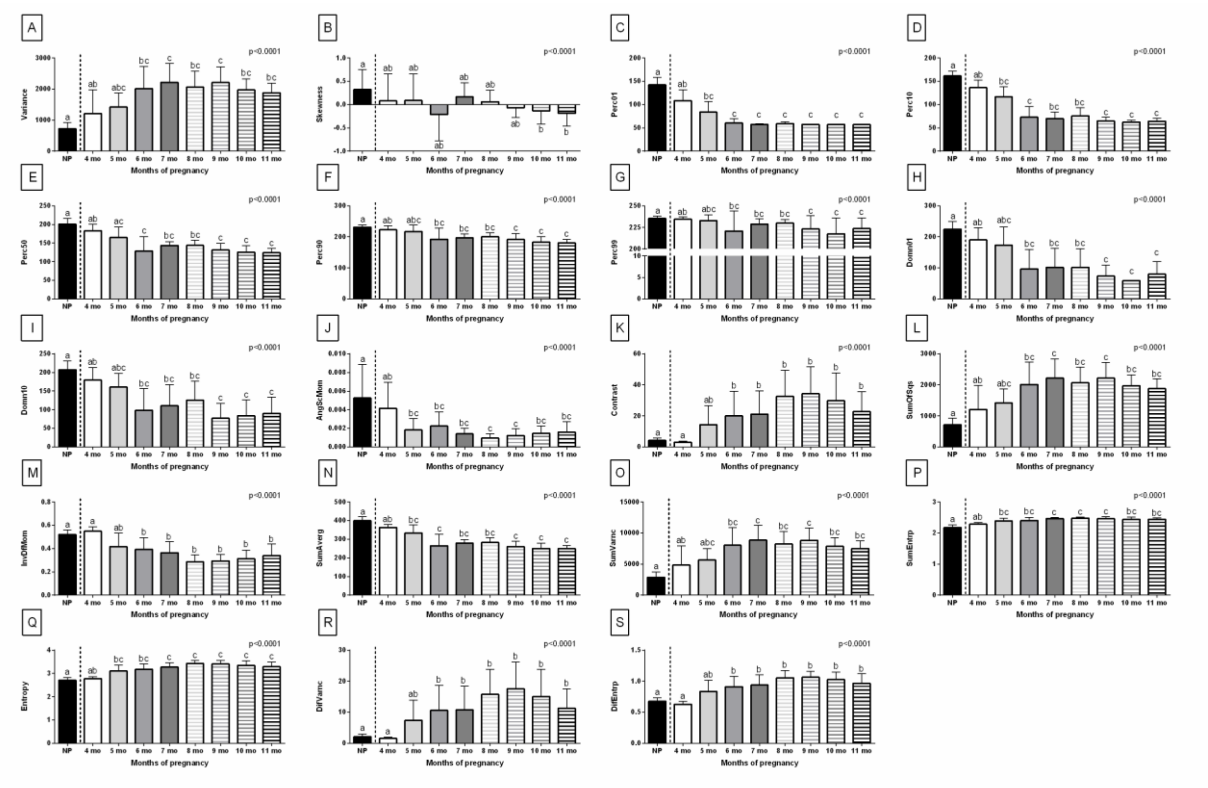

Figure S6. Nineteen selected features (mean +SD) of the Histogram Statistics and Gray Level Co-occurrence Matrix for the V-component in the YUV color model compared between the non-pregnant group and consecutive pregnant group over consecutive months of pregnancy. Differences between groups were indicated with individual p-value when p<0.05. Different superscripts within pregnant (a, b, c) were statistically different. Consecutive plots represent (**A**) Variance; (**B**) Skewness - skewness coefficient; (**C**) Perc01 - percentile 01; (**D**) Perc10 - percentile 10; (**E**) Perc50 - percentile 50; (**F**) Perc90 - percentile 90; (**G**) Perc99 - percentile 99; (**H**) Domn01 - dominant 01; (**I**) Domn10 - dominant 10; (**J**) AngScMom - angular second moment/energy; (**K**) Contrast; (**L**) SumOfSqs - sum of squares; (**M**) InvDefMom - inverse different moment/homogeneity; (**N**) SumAverg - summation mean; (**O**) SumVarnc - summation variance; (**P**) SumEntrp - summation entropy; (**Q**) Entropy; (**R**) DifVarnc - differential variance; (**S**) DifEntrp - differential entropy.

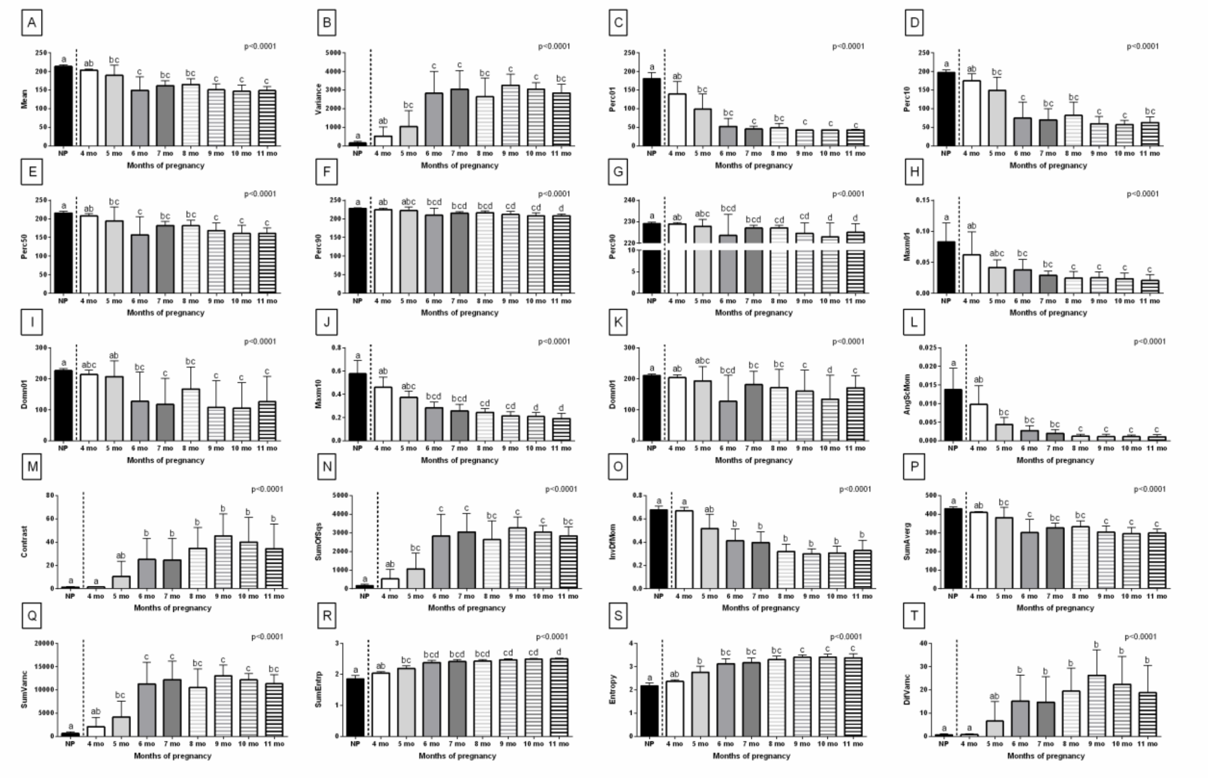

Figure S7. Twenty selected features (mean +SD) of the Histogram Statistics and Gray Level Co-occurrence Matrix for the I-component in the YIQ color model compared between the non-pregnant group and consecutive pregnant group over consecutive months of pregnancy. Differences between groups were indicated with individual p-value when p<0.05. Different superscripts within pregnant (a, b, c, d) were statistically different. Consecutive plots represent (A) Mean; (B) Variance; (C) Perc01 - percentile 01; (D) Perc10 - percentile 10; (E) Perc50 - percentile 50; (F) Perc90 - percentile 90; (**G**) Perc99 - percentile 99; (**H**) Maxm01 - maximum of moment 01; (**I**) Domn01 - dominant 01; (**J**) Maxm10 - maximum of moment 10; (**K**) Domn10 - dominant 10; (**L**) AngScMom - angular second moment/energy; (**M**) Contrast; (**N**) SumOfSqs - sum of squares; (**O**) InvDefMom - inverse different moment/homogeneity; (**P**) SumAverg - summation mean; (**Q**) SumVarnc - summation variance; (**R**) SumEntrp - summation entropy; (**S**) Entropy; (**T**) DifVarnc - differential variance.

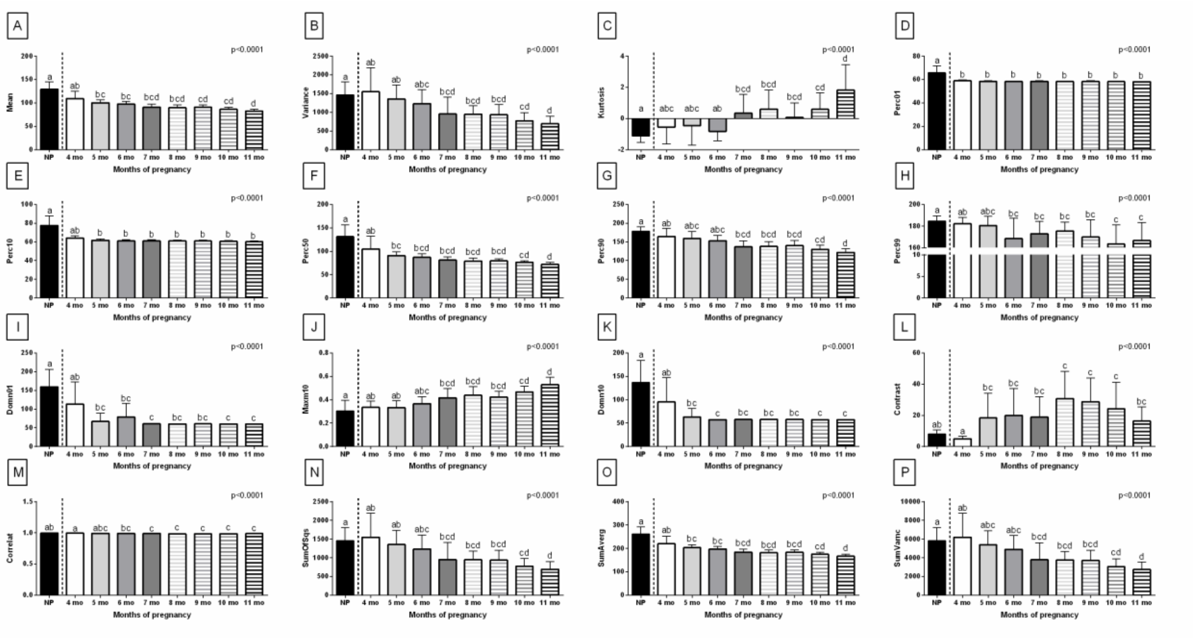

Figure S8. Sixteen selected features (mean +SD) of the Histogram Statistics and Gray Level Co-occurrence Matrix for the Q-component in the YIQ color model compared between the non-pregnant group and consecutive pregnant group over consecutive months of pregnancy. Differences between groups were indicated with individual p-value when p<0.05. Different superscripts within pregnant (a, b, c, d) were statistically different. Consecutive plots represent (**A**) Mean; (**B**) Variance; (**C**) Kurtosis; (**D**) Perc01 - percentile 01; (**E**) Perc10 - percentile 10; (**F**) Perc50 - percentile 50; (**G**) Perc90 - percentile 90; (**H**) Perc99 - percentile 99; (**I**) Domn01 - dominant 01; (**J**) Maxm10 - maximum of moment 10; (**K**) Domn01 - dominant 01; (**L**) Contrast; (**M**) Correlat - correlation; (**N**) SumOfSqs - sum of squares; (**O**) SumAverg - summation mean; (**P**) SumVarnc - summation variance.

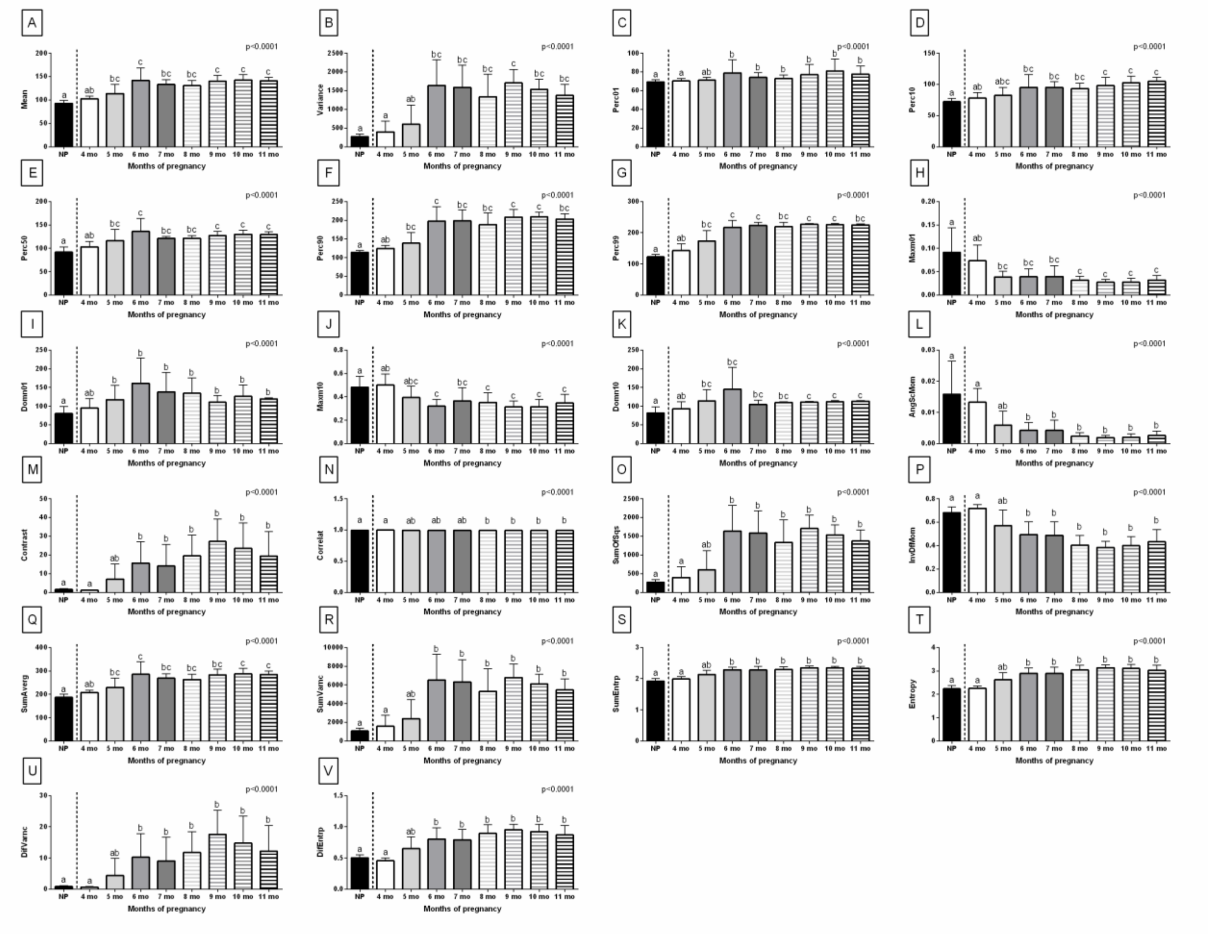

Figure S9. Twenty two selected features (mean +SD) of the Histogram Statistics and Gray Level Co-occurrence Matrix for the Hue component in the HSB color model compared between the non-pregnant group and consecutive pregnant group over consecutive months of pregnancy. Differences between groups were indicated with individual p-value when p<0.05. Different superscripts within pregnant (a, b, c) were statistically different. Consecutive plots represent (**A**) Mean; (**B**) Variance; (**C**) Perc01 - percentile 01; (**D**) Perc10 - percentile 10; (**E**) Perc50 - percentile 50; (**F**) Perc90 - percentile 90; (**G**) Perc99 - percentile 99; (**H**) Maxm01 - maximum of moment 01; (**I**) Domn01 - dominant 01; (**J**) Maxm10 - maximum of moment 10; (**K**) Domn10 - dominant 10; (**L**) AngScMom - angular second moment/energy; (**M**) Contrast; (**N**) Correlat - correlation; (**O**) SumOfSqs - sum of squares; (**P**) InvDefMom - inverse different moment/homogeneity; (**Q**) SumAverg - summation mean; (**R**) SumVarnc - summation variance; (**S**) SumEntrp - summation entropy; (**T**) Entropy; (**U**) DifVarnc - differential variance; (**V**) DifEntrp - differential entropy.

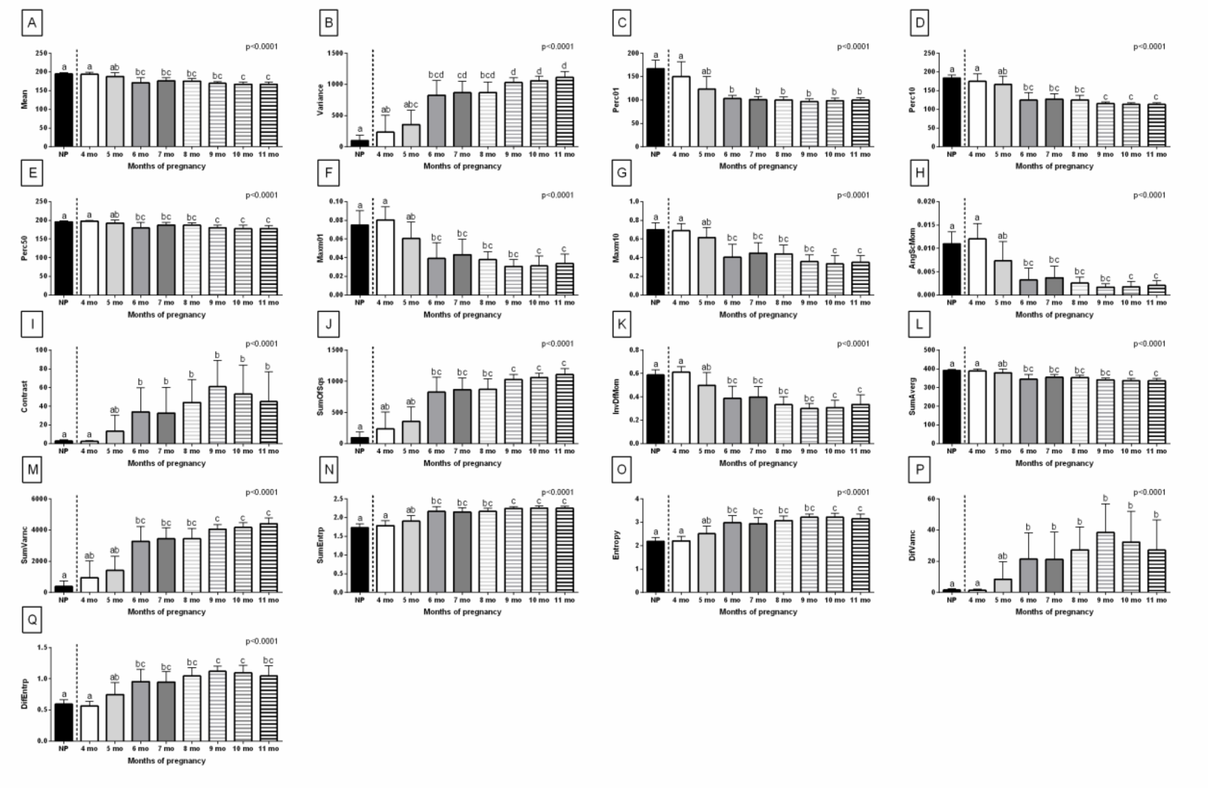

Figure S10. Seventeen selected features (mean +SD) of the Histogram Statistics and Gray Level Co-occurrence Matrix for the Saturation component in the HSB color model compared between the non-pregnant group and consecutive pregnant group over consecutive months of pregnancy. Differences between groups were indicated with individual p-value when $p<0.05$. Different superscripts within pregnant (a, b, c, d) were statistically different. Consecutive plots represent (**A**) Mean; (**B**) Variance; (**C**) Perc01 - percentile 01; (**D**) Perc10 - percentile 10; (**E**) Perc50 - percentile 50; (**F**) Maxm01 - maximum of moment 01; (**G**) Maxm10 - maximum of moment 10; (**H**) AngScMom - angular second moment/energy; (**I**) Contrast; (**J**) SumOfSqs - sum of squares; (**K**) InvDefMom - inverse different moment/homogeneity; (**L**) SumAverg - summation mean; (**M**) SumVarnc - summation variance; (**N**) SumEntrp - summation entropy; (**O**) Entropy; (**P**) DifVarnc - differential variance; (**Q**) DifEntrp - differential entropy.
